# Deletion of Growth Hormone Secretagogue Receptor in Kisspeptin Neurons in Female Mice Blocks Diet-Induced Obesity

**DOI:** 10.3390/biom12101370

**Published:** 2022-09-25

**Authors:** Kristie Conde, Danielle Kulyk, Allison Vanschaik, Sierra Daisey, Catherine Rojas, Kimberly Wiersielis, Ali Yasrebi, Thomas J. Degroat, Yuxiang Sun, Troy A. Roepke

**Affiliations:** 1 Graduate Program in Neuroscience, Rutgers University Robert Wood Johnson Medical School, The State University of New Jersey, New Brunswick, NJ 08901, USA; 2 Department of Animal Sciences, School of Environmental and Biological Sciences, Rutgers, The State University of New Jersey, New Brunswick, NJ 08901, USA; 3 Joint Graduate Program in Toxicology, Rutgers, The State University of New Jersey, Piscataway, NJ 08854, USA; 4 Department of Nutrition, College of Agriculture and Life Sciences, Texas A&M University, College Station, TX 77843, USA; 5 Environmental and Occupational Health Sciences Institute, Rutgers, The State University of New Jersey, Piscataway, NJ 08854, USA; 6 Rutgers Center for Lipid Research, the Center for Nutrition, Microbiome, and Health, and the New Jersey Institute of Food, Nutrition, and Health, Rutgers, The State University of New Jersey, New Brunswick, NJ 08901, USA

**Keywords:** Kisspeptin, 17β-estradiol, ghrelin, GHSR, reproduction, energy homeostasis

## Abstract

The gut peptide, ghrelin, mediates energy homeostasis and reproduction by acting through its receptor, growth hormone secretagogue receptor (GHSR), expressed in hypothalamic neurons in the arcuate (ARC). We have shown 17β-estradiol (E2) increases *Ghsr* expression in Kisspeptin/Neurokinin B/Dynorphin (KNDy) neurons, enhancing sensitivity to ghrelin. We hypothesized that E2-induced *Ghsr* expression augments KNDy sensitivity in a fasting state by elevating ghrelin to disrupt energy expenditure in females. We produced a Kiss1-GHSR knockout to determine the role of GHSR in ARC KNDy neurons. We found that changes in ARC gene expression with estradiol benzoate (EB) treatment were abrogated by the deletion of GHSR and ghrelin abolished these differences. We also observed changes in metabolism and fasting glucose levels. Additionally, knockouts were resistant to body weight gain on a high fat diet (HFD). Behaviorally, we found that knockouts on HFD exhibited reduced anxiety-like behavior. Furthermore, knockouts did not refeed to the same extent as controls after a 24 h fast. Finally, in response to cold stress, knockout females had elevated metabolic parameters compared to controls. These data indicate GHSR in Kiss1 neurons modulate ARC gene expression, metabolism, glucose homeostasis, behavior, and thermoregulation, illustrating a novel mechanism for E2 and ghrelin to control Kiss1 neurons.

## 1. Introduction

Rodents have been known to exhibit torpor during periods of food scarcity to compensate for a negative energy balance via suppression of the hypothalamic-pituitary-gonadal (HPG) axis [1]. These periods induce a state of hypothermia and a reduction in metabolism that allow for survival. During these sensitive periods, reproduction becomes secondary to conserving energy. There are numerous neuroendocrine mechanisms that link reproduction with metabolism and thermoregulation; however, they are not well understood in most mammals. Ghrelin is one peripheral hormone that could link the neuromodulation of reproduction and metabolism during times of food scarcity [2,3,4]. Ghrelin is produced primarily by the stomach to drive feeding via the ghrelin receptor, growth hormone secretagogue receptor (GHSR), which is expressed in hypothalamic arcuate (ARC) neuropeptide Y (NPY) and KNDy (Kisspeptin-Neurokinin B-Dynorphin) neurons [5,6]. Ghrelin is known to suppress luteinizing hormone (LH) pulse frequency in rodents, through β-endorphin signaling from proopiomelanocortin (POMC) neurons, even though less than 10% of POMC neurons express GHSR [7,8,9,10,11]. Importantly, arcuate KNDy neurons project to both NPY and POMC neurons within the ARC and receive reciprocal projections [12].

In rodent models, reproduction is controlled by the negative and positive feedback of 17β-estradiol (E2) on the HPG axis, mediated by neurons expressing kisspeptin in the ARC and the anteroventral periventricular (AVPV) nucleus, respectively [13]. AVPV kisspeptin neurons respond to high concentrations of E2 to stimulate the GnRH surge into the portal vein system of the median eminence to control the surge of luteinizing hormone (LH) necessary for ovulation. Both AVPV and ARC kisspeptin neurons control the tonic release of LH and follicle stimulating hormone (FSH) production, essential for both gametogenesis and steroidogenesis. Neurokinin B and dynorphin in KNDy neurons act as positive and negative autoregulators of KNDy neuronal excitability, respectively, to produce the kisspeptin-regulated pulse generator that controls tonic GnRH release [14,15,16,17,18]. Kisspeptin controls GnRH release by binding to its receptor Kiss1R expressed on the soma and axons of GnRH neurons [19,20].

While ghrelin actions on NPY neurons and in energy homeostasis are well characterized [5], only a few studies have examined the actions of ghrelin on KNDy neurons. One study showed ARC Kiss1 neurons co-expressed GHSR and acute actions of ghrelin on ARC Kiss1 neurons are modulated by estradiol, showing greater depolarization in response to ghrelin with E2 administration [21]. KNDy neurons also contribute to the control of energy homeostasis and thermoregulation in females [22]. Ablation of KNDy neurons blocks the effects of E2 on post-ovariectomy weight gain in rats, suggesting that KNDy neurons mediate, in part, the anorectic effects of E2 [23]. One pathway for KNDy neurons to control energy balance is by directly depolarizing POMC neurons through kisspeptin and glutamate release [24,25]. Furthermore, recent thermoregulation studies have confirmed that neurokinin B, from ARC KNDy neurons, mediates hot flushes in response to reduced E2 [26,27].

E2, a primary ovarian steroid hormone, also plays a critical role in regulating reproduction [28,29,30]. One of many mechanisms for E2 to link these processes is by modulating hormone receptor expression in hypothalamic neurons, like KNDy neurons, that also respond to negative feedback of E2 [12,31]. For example, we have observed that E2 increases *Ghsr* expression in arcuate KNDy neurons by 6-fold [31]. After further exploration, using whole-cell patch-clamp electrophysiology, we confirmed that when E2 is high, the increased *Ghsr* expression enhances KNDy sensitivity to ghrelin via inhibition of the M-current [32]. E2 reduces food intake and increases energy expenditure and activity, in part, through actions in the mediobasal hypothalamus [33,34,35,36]. KNDy neurons modulate this circuit by simultaneously exciting and inhibiting POMC and NPY neurons, respectively, through glutamatergic signaling in both male and female mice [12,24]. Therefore, while high E2 levels suppress *Kiss1* gene expression, E2 augments KNDy *Ghsr* expression [31], increasing ghrelin sensitivity [32], and stimulating glutamate release, leading to differential modulation of the arcuate melanocortin circuit [37]. This ghrelin-induced KNDy activity reinforces the anorectic actions of E2 by exciting POMC tone and reducing ghrelin’s activation of NPY/AgRP neurons.

In summary, the arcuate neuronal circuit of POMC, NPY/AgRP, and KNDy neurons regulate reproduction, energy balance, and thermoregulation by responding to circulating nutrients, gonadal and adrenal steroids, and appetite-regulating hormones such as ghrelin [38]. We hypothesize that the ghrelin-induced KNDy activity may reinforce the actions of E2 by elevating POMC tone and reducing ghrelin-induced NPY/AgRP activity to regulate sympathetic output that controls metabolism and thermogenesis, while also suppressing LH pulsatility through β-endorphin release during states of elevated ghrelin in females [12,37]. To address this hypothesis, we developed a novel transgenic mouse model that selectively deleted GHSR in Kiss1-expressing cells and measured reproductive, metabolic, and behavioral parameters.

## 2. Materials and Methods

### 2.1. Animals

All animal procedures were completed in compliance with institutional guidelines based on National Institutes of Health standards and were performed with Institutional Animal Care and Use Committee approval at Rutgers University. Adult mice were housed under constant photoperiod conditions (12/12 h light/dark cycle) and maintained at a controlled temperature (25 °C). Animals were given food and water ad libitum, unless noted otherwise. Animals were weaned at postnatal day 21 (PD21). Sexually mature *Tac2*-EGFP male mice, used previously [15,31,32], were used for cell harvesting experiments (Figures S1 and S2). 

### 2.2. Production of Kiss1^Cre/EGFP^;Ghsr^fl/fl^ Mice

To produce a selective deletion of GHSR in *Kiss1* neurons, we mated C57BL/6J- Kiss11tm1.1(cre/EGFP)Stei/J mice from Jackson Laboratory (#017701) to a floxed GHSR (GHSR^fl/fl^). The GHSR^fl/fl^ mouse strain provided by Dr. Yuxiang Sun (Texas A&M) were backcrossed over 10 generations. The Kiss1Cre/EGFP allele expresses a Cre-EGFP fusion protein from the *Kiss1* promoter and enhancer elements. This Cre line has been used to develop other *Kiss1*-specific knockouts and knockins, as well as determine the molecular signaling properties of Kiss1 neurons and their role in GnRH, LH, AgRP, and POMC signaling [12,37,39,40,41,42,43,44,45]. Cre-mediated recombination will result in deletion of GHSR from *Kiss1*-expressing neurons in offspring (Kiss1^Cre/+^/Ghsr^fl/fl^). Littermates that did not express the Kiss1^Cre^/EGFP allele (Kiss1^WT^/Ghsr^fl/fl^) were used as controls. Control and Kiss1-GHSR KO (knockout) females were used for all experiments, unless noted otherwise. We have verified the decrease in GHSR expression in ARC *Kiss1* neurons by analyzing pools of *Kiss1* neurons from *Tac2*-GFP females (+*Ghsr* expression) and Kiss1^Cre/+^/Ghsr^fl/fl^ females (-*Ghsr* expression) (See Supplemental Methods for Cell Harvesting protocol and Figure S1) [46]. Only females were used in this study as we did not detect *Ghsr* in KNDy neurons in *Tac2*-GFP male mice using single cell RT-PCR nor did testosterone propionate increase *Ghsr* expression in these neurons using quantitative real-time PCR (See Supplemental Methods Cell Harvesting protocol and Figure S2).

### 2.3. Chemicals

For non-energy homeostasis experiments, an estradiol benzoate (EB) injection protocol was used that has previously been shown to alter gene expression in the hypothalamus and mimic a proestrus-like state in mice [20]. Briefly, animals were injected subcutaneously (s.c.) at 10:00 am on post-OVX/GDX day 5 with either 0.25 μg of EB or sesame oil-vehicle. On post-OVX/GDX day 6, a 1.5 μg dose of EB or sesame oil-vehicle was injected at 10:00 am. Ghrelin was purchased from AnaSpec Peptides (Fremont, CA, USA) and was dissolved in saline prior to storage at −20 °C until needed. Estradiol benzoate (EB) was purchased from Steraloids (Newport, RI, USA). Ketamine, Marcaine, and Rimadyl were purchased from Henry Schein Animal Health (Dublin, OH, USA). EB was dissolved in 100% ethanol prior to suspension in sesame oil. Ethanol was allowed to evaporate for 24 h prior to storage at 4 °C until needed. For energy balance experiments, diets were purchased from Research Diets (New Brunswick, NJ, USA): low-fat diet (LFD; 10% kcal fat; D12450B) and high-fat diet (HFD; 45% kcal fat; D12451).

### 2.4. RNA Extraction and Quantitative Real-Time PCR

To determine if ghrelin alters ARC gene expression (Experiment #1), Kiss1-GHSR KO and control mice were separated into 4 treatment groups: (1) OVX/Oil + ghrelin (12 h prior), (2) OVX/EB + ghrelin, (3) OVX/Oil, and (4) OVX/EB. Collection of tissues, RNA extraction, and qPCR protocols were the same as previously published [47,48,49]. Briefly, brain slices were collected using a brain matrix (Ted Pella, Redding, CA, USA) which allows for the cutting of the brain into 1 mm-thick coronal slices as previously described [50]. Brain slices containing the region of interest were transferred to RNALater (Life Technologies) and stored overnight at 4 °C. For model validation, in addition to the ARC, we also performed RNA extraction using the same technique on tissues including: Anteroventral Paraventricular Nucleus (AVPV), Bed Nucleus of Striata Terminalis (BNST), medial Amygdala (AMG), Ventral Tegmental Area (VTA), and Substantia Nigra (SubN; Figures S6–S8).

Pure RNA was extracted from all brain tissue using Ambion RNAqueous^®^ Micro Kits (Life Technologies, Carlsbad, CA, USA) according to the manufacturer’s protocol, followed by DNase-I treatment to remove contamination by genomic DNA (Life Technologies). Quantity of RNA was assessed by the NanoDrop™ ND-2000 spectrophotometer (ThermoFisher, Inc., Waltham, MA, USA), followed by the RNA 6000 Nano Kit (Agilent Technologies, Inc., Santa Clara, CA, USA) to assess quality. Samples with an RNA integrity number (RIN) greater than 8 were used for quantitative real-time PCR (qPCR). As previously described [47] complementary DNA (cDNA) was synthesized from 250 ng of total RNA using Superscript III reverse transcription (Life Technologies). cDNA was diluted to 1:20 with nuclease-free water (Gene Mate/Bioexpress) for a final cDNA concentration of 0.5 ng/μL and stored at −20 °C. Untreated ARC tissue RNA was used for the calibrator and negative control (no reverse transcription) and processed simultaneously with the Kiss1-GHSR KO samples. All values were normalized and are expressed as relative mRNA expression. Efficiencies were calculated as a percent efficiency and are approximately equal (90–110% or one doubling per cycle, see Table 1 for a list of primer sequences). The reference genes used were *Gapdh* and *Hprt*. Positive, negative, and water blank controls were included in the qPCR plate design. The geomean of the Cq values from each reference gene was used to calculate relative gene expression, reference gene Cq values were not affected by the treatments [47,49]. Average Cq values are presented in Table S1. 

### 2.5. Surgical Procedures

Adult females were bilaterally ovariectomized (OVX) under isoflurane anesthesia using a previously published sterile no-touch technique according to the NIH Guidelines for Survival Rodent Surgery [31,32,51]. Animals were given a dose of analgesic [4 mg/kg carprofen (Rimadyl^®^)] one day following surgery for pain management. Animals typically lost 1–2 g of weight one day after surgery, which was regained by post-surgery day three.

For energy homeostasis experiments (Experiment #2), following OVX, females were separated into two treatment groups–sesame oil vehicle and estradiol benzoate (EB). To reduce constant stressful injections during the 8 weeks of diet, mice were passively orally dosed with sesame oil vehicle or EB (300 μg/kg body weight; suspended in sesame oil) mixed with powdered, peanut butter daily [31]. After sacrifice, uteri were weighed to confirm effect of treatment.

### 2.6. Food Intake, Body Weight, and Body Composition

To determine the influence of E2 (Experiment #2) and high-fat diet (Experiment #3) on the interaction of Kiss1 and GHSR, food intake and body weight were measured weekly. Body composition was measured using a small rodent MRI (EchoMRI, Houston, Texas). For experiment #2, females were OVX and received EB- or oil-treatment. For experiment #3, females remained gonadally intact.

### 2.7. Metabolic Monitoring

To determine the influence of E2 (Experiment #2) and high-fat diet (Experiment #3) on the interaction of Kiss1 and GHSR and metabolic outcomes, indirect calorimetry, food intake, and activity were measured using CLAMS chambers (Comprehensive Lab Animal Monitoring System, Columbus Instruments, Columbus, OH, USA). Mice were in the CLAMS chambers for 4 days total, the first 2 days of which were an acclimation period and the final 48 h were used for data analysis.

### 2.8. Glucose and Insulin Tolerance Tests

To determine the influence of E2 (Experiment #2) and high-fat diet (Experiment #3) on the interaction of Kiss1 and GHSR and glucose homeostasis, a glucose tolerance test (GTT) and insulin tolerance test (ITT) were performed a minimum of 4 days apart in the same animals. Each mouse received an intraperitoneal (IP) injection of glucose (2 g/kg) after a 5 h fast (09:00 am–02:00 pm) or insulin (0.75 U/kg body weight in sterile saline) after a 4 h fast (09:00 am–01:00 pm). Glucose was measured in tail blood using an AlphaTrak glucometer (Zoetis, Parsippany, NJ, USA). Glucose measurements were taken at 0, 15, 30, 60, 90, and 120 min after injection. Fasting blood glucose was determined from the baseline timepoint during GTT, before glucose administration.

### 2.9. Behavioral Tests

To determine the influence of high-fat diet on the interaction of Kiss1 and GHSR and locomotor and anxiety-like behavior (Experiment #4), we performed behavioral tests commonly used to assess avoidance behaviors, protocols used were previously published [51]. Tests were performed in the same mice with a 5 to 7 day rest period in between.

#### 2.9.1. Open Field Test (OFT)

The OFT evaluates anxiety-like behavior in concert with spontaneous exploratory activity [52]. Mice were first subjected to the open field test consisting of a square white opaque arena area (40 cm long × 40 cm wide × 40 cm high, open-top) with a 64-square grid floor (8 × 8 squares, 5 cm/side). Each mouse was placed in the same (10 cm long × 10 cm wide) corner square of the arena and allowed to freely explore for 10 min. After each animal trial, the surface of the arena was cleaned with a sterilizing solution.

#### 2.9.2. Elevated Plus-Maze (EPM)

Mice were next assessed on the EPM. The EPM assesses anxiety-like behavior and is often used in combination with the OFT [53]. The apparatus consisted of two sets of opposing arms (30 cm × 5 cm) extending from a central (5 cm × 5 cm) region. Two arms were enclosed (15 cm high walls) and two arms remained open. Animals were placed in the central area of the maze in the direction of a closed arm and allowed to freely explore both open and closed arms for 5 min. Following each animal trial, the surface of the arena was cleaned with a sterilizing solution.

#### 2.9.3. Light/Dark Box (LDB)

The LDB is another task that evaluates anxiety-like behavior and spontaneous exploratory activity, but through a natural conflict situation between innate exploratory desire of novelty and tendency to avoid the unfamiliar [54]. The LDB assessment took place using a square white opaque arena (40 cm long × 40 cm wide × 40 cm high, open-top), with a black rectangle insert (40 cm long × 20 cm wide × 40 cm high, closed top) that contained an opening (7.5 cm × 7.5 cm) located at floor level in the center of the partition. Each mouse was placed in the same corner of the light side of the apparatus and allowed to freely explore for 10 min. After each animal trial, the surface of the arena was cleaned with a sterilizing solution.

### 2.10. The Effects of Fasting on Meal Patterns

To determine the influence of fasting (Experiment #5) on the interaction of Kiss1 and GHSR and feeding behavior, Kiss1-GHSR KO and control females were placed in the biological data acquisition system (BioDAQ; Biological Data Acquisition, Research Diets, New Brunswick, NJ, USA). Mice were fed a normal chow diet and were allowed to acclimate to single housing for 4 days followed by a 3-day acclimation to the BioDAQ. After acclimation, 1 h before lights out, half of the mice were fasted for 24 h and the other half were fed ad lib. After 24 h, food was returned to the fasted mice and meal patterns were recorded, uninterrupted for the next 48 h.

### 2.11. Core Body Temperature Recording 

To determine the influence of mild cold stress (Experiment #6) on the interaction of Kiss1 and GHSR, core body temperature was recorded using temperature loggers (DST nano, Star-Oddi, HerfØlge, Denmark) implanted abdominally, utilizing similar methods as previously published [55]. Loggers were placed in the abdominal cavity using similar surgical technique as OVX and recorded temperature every five minutes until removed. After a 1-week recovery from surgery, mice were placed into the CLAMS and acclimated to the chambers for 3 days. After acclimation, recording began at 25 °C to access baseline. During the second night, the environmental temperature was reduced from 25 °C to 10 °C for a period of 6 h. Mice remained inside the CLAMS for an additional night at 25 °C. At the end of the experiments, the loggers were retrieved, and the data were downloaded and analyzed.

### 2.12. Statistical Analysis

All data were analyzed by a 2-way ANOVA (genotype and steroid/diet) followed by a post hoc Holm–Sidak’s except for q-PCR data which were analyzed by multifactorial ANOVA (genotype and steroid) using Statistica (Dell, Round Rock, TX, USA). All gene expression data were normalized to control females for comparison across genotypes. For the behavioral studies, values that exceeded 2 SDs above or below the group mean were considered outliers and dropped. LH values (see supplemental methods) were analyzed by two-tailed unpaired Student t-test to compare LH pulse parameters between Kiss1-GHSR KO females in proestrus and diestrus, Kiss1-GHSR KO and control females in diestrus after a 24 h fast, Kiss1-GHSR KO females in diestrous with and without ghrelin, OVX Kiss1-GHSR KO females treated with EB or oil, OVX Kiss1-GHSR KO females treated with EB and treated with EB/ghrelin, and OVX/EB treated control and Kiss1-GHSR KO females with ghrelin. Results were considered statistically significant at *p* < 0.05.

## 3. Results

### 3.1. Experiment #1: Regulation of ARC Gene Expression

**Figure 1:**
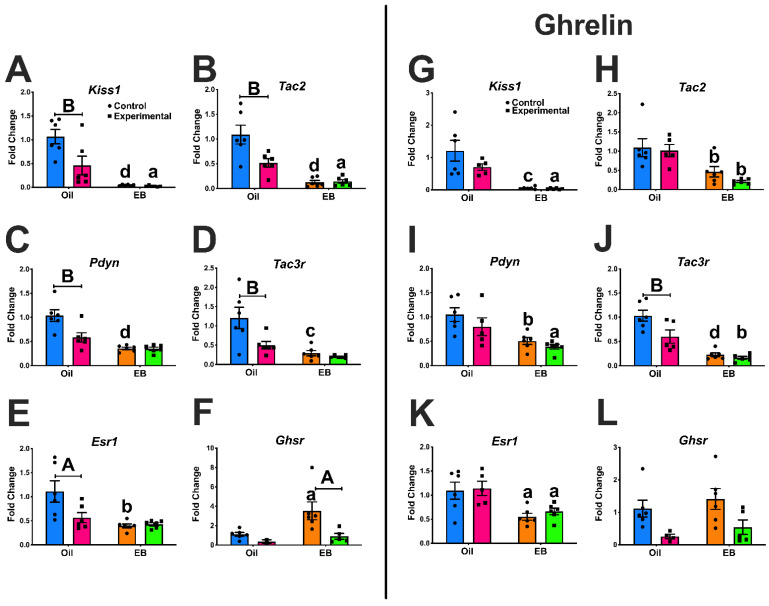
Arcuate gene expression in OVX control and Kiss1-GHSR KO females with or without E2 replacement (**A**–**F**) and with ghrelin injection (**G**–**L**): (**A**,**G**) *Kiss1*; (**B**,**H**) *Tac2*; (**C**,**I**) *Pdyn*; and (**D**,**J**) *Tac3r*; (**E**,**K**) *Esr1*; and (**F**,**L**) *Ghsr*. For all graphs, data were analyzed by a multi-factorial ANOVA (steroid, genotype) with post hoc Holm–Sidak’s multiple comparison test. Lower case letters denote steroid differences within genotype, uppercase letters above capped lines denote genotype effects (A/a = *p* < 0.05; B/b = *p* < 0.01; C/c = *p* < 0.001; D/d = *p* < 0.0001). Data is represented as mean ± SEM. Ghrelin injection was given IP at 1 mg/kg 12 h prior to sacrifice. Statistics are indicated as follows: *Kiss1* (**A**): steroid: F(1,20) = 34.34; genotype: F(1,20) = 6.385; interaction of steroid*genotype: F(1,20) = 5.532. *Tac2* (**B**): steroid: F(1,20) = 39.06; genotype: F(1,20) = 6.661; interaction of steroid*genotype: F(1,20) = 7.573. *Pdyn* (**C**): steroid: F(1,20) = 32.32; genotype: F(1,20) = 8.140; interaction of steroid*genotype: F(1,20) = 7.674. *Tac3r* (**D**): steroid: F(1,20) = 16.43; genotype: F(1,20) = 7.042. With ghrelin, *Kiss1* (**G**): steroid: F(1,19) = 27.39. *Tac2* (**H**): steroid: F(1,19) = 20.43. *Pdyn* (**I**): steroid: F(1,19) = 16.72. *Tac3r* (**J**): steroid: F(1,19) = 50.60; genotype: F(1,19) = 7.924, interaction of steroid*genotype: F(1,19) = 4.457. *Esr1* (**K**): steroid: F(1,19) = 16.83. *Ghsr* (**L**): genotype: F(1,17) = 10.53.

To determine the interactions of E2 and ghrelin on ARC gene expression without *Ghsr* expression in *Kiss1* neurons, we focused on E2-responsive genes that are involved in energy homeostasis and reproduction. In Figure 1, expression of neuropeptide KNDy genes *Kiss1* (Figure 1A; steroid: *p* < 0.0001; genotype: *p* = 0.0201; interaction of steroid*genotype: *p* = 0.0290), *Tac2* (Figure 1B; steroid: *p* < 0.0001; genotype: *p* = 0.0178; interaction of steroid*genotype: *p* = 0.0123), *Pdyn* (Figure 1C; steroid: *p* < 0.0001; genotype: *p* = 0.0098; interaction of steroid*genotype: *p* = 0.0118), and *Tac3r* (Figure 1D; steroid: *p* = 0.0006; genotype: *p* = 0.0152) were reduced by EB in control females. Furthermore, EB reduced *Esr1* gene expression (Figure 1E; steroid: *p* = 0.0028; interaction of steroid*genotype: *p* = 0.0323) and elevated *Ghsr* expression (Figure 1F; genotype: *p* = 0.0290) in controls. In contrast, in Kiss1-GHSR KO females, gene expression was only reduced for *Kiss1* (Figure 1A; *p* < 0.05) and *Tac2* (Figure 1B; *p* < 0.05) while *Pdyn*, *Tac3r*, Esr1, and *Ghsr* remained unchanged by EB treatment. In addition, oil-treated Kiss1-GHSR KO females revealed reduced gene expression for *Kiss1* (Figure 1A; *p* < 0.01), *Tac2* (Figure 1B; *p* < 0.01), *Pdyn* (Figure 1C; *p* < 0.01), *Tac3r* (Figure 1D; *p* < 0.01), and *Esr1* (Figure 1E; *p* < 0.05), compared to controls, while *Ghsr* expression was increased by EB-treatment only in controls (Figure 1F; *p* < 0.05) and EB-treated Kiss1-GHSR KO females showed reduced *Ghsr* expression compared to oil-treated (Figure 1F; *p* < 0.05). These data confirm that the E2-induced increase in ARC *Ghsr* expression is likely due to up-regulation in *Kiss1* neurons [31].

To determine the interaction of E2 and ghrelin on ARC gene expression, we repeated the same experiment with the addition of a ghrelin injection (1 mg/kg; IP), given 12 h prior to sacrifice [56]. In Figure 1, expression of neuropeptide KNDy genes *Kiss1* (Figure 1G; steroid: *p* < 0.0001), *Tac2* (Figure 1H; steroid: *p* = 0.0002), *Pdyn* (Figure 1I; steroid: *p* = 0.0006), and Tac3r (Figure 1J; steroid: *p* < 0.0001; genotype: *p* = 0.0111, interaction of steroid*genotype: *p* = 0.0482) were reduced by EB and ghrelin in control females. Furthermore, EB reduced *Esr1* expression (Figure 1K; steroid: *p* = 0.0006) and abrogated the change in *Ghsr* expression (Figure 1L; genotype: *p* = 0.0048) in controls. In contrast to gene expression without ghrelin (Figure 1A–F), in Kiss1-GHSR KO females treated with EB and ghrelin, gene expression reduction was expanded beyond *Kiss1* (Figure 1G; steroid: *p* < 0.0001), and *Tac2* (Figure 1H; steroid: *p* = 0.0002) to include *Pdyn* (Figure 1I; steroid: *p* = 0.0006), *Tac3r* (Figure 1J; steroid: *p* < 0.0001; genotype: *p* = 0.0111; interaction of genotype*steroid: *p* = 0.0482), and *Esr1* (Figure 1K; steroid: *p* = 0.0006), while *Ghsr* remained unchanged (Figure 1L; genotype: *p* = 0.0048). In addition, there were only genotype differences in oil and ghrelin-treated Kiss1-GHSR KO females for *Tac3r* (Figure 1J), while ghrelin injection abrogated genotype differences seen previously with *Kiss1*, *Tac2*, *Pdyn* and *Esr1*. In addition, we examined ARC *Npy*, *Agrp*, *Pomc*, and *Cart* gene expression in the same experimental design revealing EB treatment enhanced *Npy* in KO females, increased *Argp* gene expression in both genotypes, reduced *Pomc* and *Cart* gene expression in control females, and reduced *Pomc* expression in KO females compared to controls treated with oil. Ghrelin administration caused an increase in *Agrp* gene expression in EB-treated controls but not KOs, while ghrelin + EB prevented an increase in *Pomc* and *Cart* expression in KO females only (Figure S7). We also analyzed gene expression comparing ghrelin-injected and non-ghrelin injected females within each genotype (Figure S4) and found a main effect of genotype in reduced *Kiss1* expression in both oil and EB-treatment (Figure S4A,B), *Tac3r* with oil-treatment (Figure S4C), *Tac2* with oil treatment (Figure S4E), *Pdyn* with oil-treatment (Figure S4I), and *Ghsr* with both oil and EB treatment (Figure S4K,L). Furthermore, we found that ghrelin injection reduced *Tac2* in oil-treated controls (Figure S4E). Finally, we found that *Tac3r*, *Tac2*, *Esr1*, and *Pdyn* were reduced in oil-treated KO females compared to controls without ghrelin (Figure S4C,E,G,I), while *Ghsr* was reduced oil-treated KO females with ghrelin compared to controls (Figure S4K) and in EB-treated KO females without ghrelin compared to controls (Figure S4L). These data indicate the importance of the interaction between EB and *Ghsr* expression when it comes to gene regulation in the ARC, specifically in Kiss1 neurons and the downstream effects.

### 3.2. Fertility and LH Pulsatility

Because ghrelin is known to suppress the frequency of pulsatile LH release in male and female rats, in part, due to activation of β-endorphin release from POMC neurons, which do not express GHSR at significant levels [7,8,9,10,11,45], we examined fertility and LH pulsatility in our females (See Supplemental Methods for Vaginal Cytology and Fertility assessments). Fertility was no different between control and KO females, the average number of days from pairing to birth was 23 ± 2 days, average litter size 6 ± 1, male to female pup ratio was equal. Vaginal opening occurred in control females at 34.5 ± 6.5 days of age and KO females were 36.5 ± 5.1 days of age. We also observed no disruption in estrous cycle. Control females were, on average in Diestrous/Metestrus for 3.54 ± 1.48 days, Proestrous for 1.63 ± 0.71 days and Estrous for 1.67 ± 0.96 days while KO females were, on average in Diestrous/Metestrus for 3.33 ± 0.98 days, Proestrous for 1.71 ± 0.58 days and Estrous for 2.04 ± 0.81 days (data not shown). LH dynamics were examined in both Kiss1-GHSR KO and control females while gonadally intact, after OVX with or without E2 replacement, and after fasting or ghrelin injection (1 mg/kg; IP) 30 min prior to the collection (See Supplemental Methods for LH Blood Collection). For intact females, there were no differences in the total number of LH peaks, LH concentration, peak amplitude, or interpulse interval. After OVX, Kiss1-GHSR KO females treated with oil had a significantly higher number of LH peaks, LH concentration and LH peak amplitude compared to EB-treated females (Figure S3).

### 3.3. Experiment #2: Response to Ovariectomy

#### 3.3.1. Body Weight Gain and Body Composition

**Figure 2:**
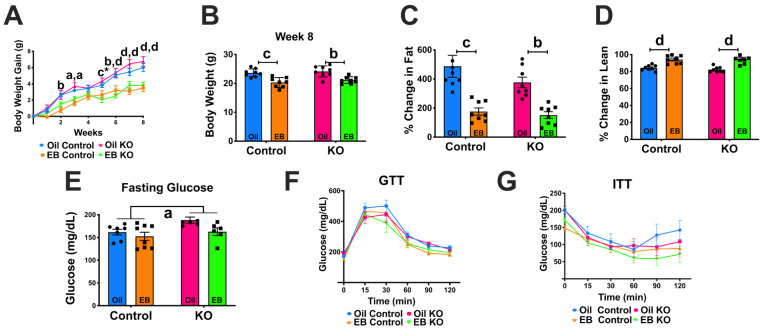
Response to ovariectomy for OVX Oil- and EB-exposed control and Kiss1-GHSR KO (KO) female mice fed LFD for 8 weeks. (**A**) Weekly body weight gain (grams), (**B**) Week 8 body weight (**C**) Percent change in fat mass, (**D**) Percent change in lean body mass (**E**) Fasting (5 h) glucose (**F**) Glucose tolerance test (GTT) and (**G**) Insulin tolerance test (ITT). For (**A**): Single lowercase letters denote a steroid effect within controls only, single lowercase letters with * denote a steroid effect within Kiss1-GHSR-KOs only, two lowercase letters denote a steroid effect in control and Kiss1-GHSR-KOs, respectively, and uppercase letters denote significance between genotypes within the same treatment. For (**B**–**D**): Capped lines with lowercase letter denote a steroid effect. For (**E**): lowercase letter under the line denotes main effect of genotype. A/a = *p* < 0.05; B/b = *p* < 0.01; C/c = *p* < 0.001; D/d = *p* < 0.0001. Data are represented as mean ± SEM, n = 8 mice per group. Data were analyzed with two-way ANOVA with post hoc Holm–Sidak’s multiple comparison test. Mice were OVX at 8 weeks of age and were 25–30 weeks of age at time of sacrifice.

EB treatment reduced body weight gain, regardless of genotype (Figure 2A; steroid: F(3,28) = 9.571, *p* = 0.0002). At the end of the 8 weeks of treatment, EB-treated females weighed less than oil-treated (Figure 2B; steroid: F(1,28) = 33.15, *p* < 0.0001). EB treatment also reduced the percent change in fat mass (Figure 2C; steroid: F(1,28) = 35.10, *p* < 0.0001) and increased the percent change in lean mass from week zero to week 8 regardless of genotype (Figure 2D; steroid: F(1,28) = 53.41, *p* < 0.0001). There were no differences observed in cumulative food intake in pair-housed females over 8 weeks of diet (data not shown). Serum concentration for Ghrelin and Insulin were measured in control and KO females treated with oil or EB (See Supplemental Methods for Serum Measurements). We found ghrelin was elevated in EB-treated-KO females compared to EB-treated controls and insulin was reduced in KO females treated with EB compared to oil (Figure S9A,B). Overall, we observed only steroid effects on body weight and body composition.

#### 3.3.2. Glucose and Insulin Tolerance Tests

Kiss1-GHSR KO females consistently exhibited elevated fasting glucose in response to a 5 h fast compared to controls, regardless of treatment (Figure 2E; F(1,23) = 5.349, *p* = 0.0300). We observed no differences in GTT or ITT (Figure 2F; time: F(5,140) = 105.65, *p* = 0.0000; Figure 2G; time: F(5,115) = 30.860, *p* = 0.0000). We observed no significant differences in area under the curve analysis of GTT or ITT (data not shown). The observed difference in fasting glucose indicates that *Ghsr* expression in Kiss1 neurons participates in the regulation of glucose homeostasis.

#### 3.3.3. Feeding, Locomotor and Metabolic Behaviors in CLAMS

**Figure 3:**
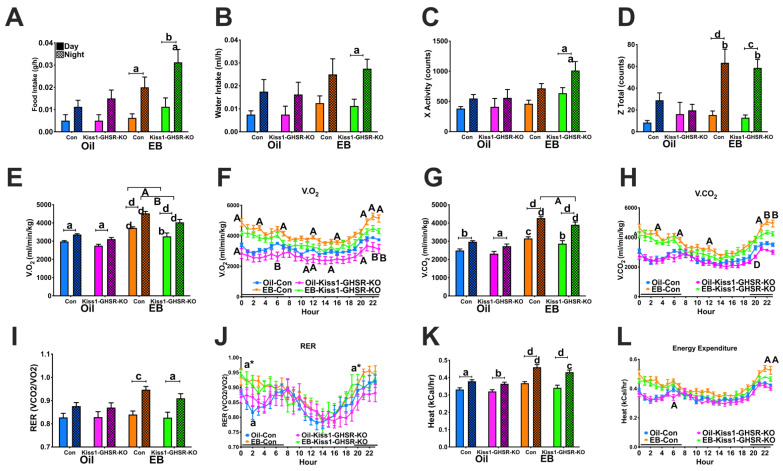
Metabolic Response to ovariectomy in OVX Oil- and EB-exposed control and Kiss1-GHSR KO (KO) female mice fed LFD for 8 weeks. (**A**) Food and (**B**) Water intake. (**C**) X-activity (counts), and (**D**) Z-activity (counts). (**E**) I Volume of oxygen consumption (V.O2, ml/min/kg), (**F**) Hourly Volume of oxygen consumption (V.O2, ml/min/kg). (**G**) Volume of carbon dioxide production (V.CO2, ml/min/kg), (**H**) Hourly volume of carbon dioxide production (V.CO2, ml/min/kg). (**I**) Respiratory exchange ratio (RER, V.CO2/V.O2), (**J**) Hourly Respiratory exchange ratio (RER, V.CO2/V.O2;), (**K**) Energy expenditure (kCal/hr) and (**L**) Hourly Energy expenditure (kCal/hr). Lowercase letters denote a steroid effect within genotype and time period, letters with * indicate a steroid effect in KO females only. Uppercase letters denote significance between genotypes within the same treatment. Lowercase letters above capped lines denote significance between time periods within treatment and genotype. A/a = *p* < 0.05; B/b = *p* < 0.01; C/c = *p* < 0.001; D/d = *p* < 0.0001. Data are represented as mean ± SEM and n = 8 mice per group. Data were analyzed with two-way ANOVA with post hoc Holm–Sidak’s multiple comparison test. Mice were 17–19 weeks of age during metabolic experiments.

EB-treated females consumed more food at night compared to the day. In addition, EB-treated controls consumed less than EB-treated Kiss1-GHSR KO females at night (Figure 3A; steroid: F(1,56) = 9.4640, *p* = 0.00324; time: F(1,56) = 22.400, *p* = 0.00002), although not significant, a main effect of genotype was trending (F(1,56) = 3.5840, *p* = 0.06351). Furthermore, water intake was elevated only in EB-treated Kiss1-GHSR KO females at night compared to the day (Figure 3B; steroid: F(1,56) = 4.9101, *p* = 0.03078; time: F(1,56) = 14.649, *p* = 0.00033). These data reveal the importance of *Ghsr* expression in Kiss1 neuronal regulation of ingestive behavior.

X-activity was increased in EB-treated Kiss1-GHSR KO females during the night compared to the day as well as compared to EB-treated controls at night (Figure 3C; steroid: F(1,56) = 10.178, *p* = 0.00233; time: F(1,56) = 10.647, *p* = 0.00188). Z-activity was similarly elevated in EB-treated females during the night compared to the day as well as compared to oil-treated females of the same genotype (Figure 3D; steroid: F(3,56) = 4.668, *p* = 0.0056; time: F(1,55) = 31.98, *p* < 0.0001; time*steroid: F(3,56) = 4.217, *p* = 0.0093). These data indicate that *Ghsr* expression in Kiss1 neurons play a role in regulating locomotor activity in female mice.

All females consumed more oxygen (V.O2) during the night compared to the day. EB-treated females consumed more oxygen during the day and night compared to oil-treated females and EB-treated controls consumed more oxygen compared to experiments during both the day and night (Figure 3E; steroid: F(1,54) = 113.10, *p* = 0.00000; genotype: F(1,54) = 21.103, *p* = 0.00003; time: F(1,54) = 54.115, *p* = 0.00000; time*steroid: F(1,54) = 6.8972, *p* = 0.01121). Hourly V.O2 consumption was elevated in EB-treated controls compared to EB-treated Kiss1-GHSR KOs at 12:00 am, 03:00 am, 07:00 am, 12:00 pm, 04:00 pm, 09:00 pm, 10:00 pm, and 11:00 pm. In addition, oil-treated controls also exhibited an increase in V.O2 compared to oil-treated Kiss1-GHSR KOs at 12:00 am, 06:00 am, 11:00 am, 12:00 pm, 03:00 pm, 08:00 pm, 10:00 pm, and 11:00 pm (Figure 3F; steroid: F(1,28) = 36.432, *p* = 0.00000; genotype: F(1,28) = 9.2728, *p* = 0.00502; time: F(23,644) = 35.816, *p* = 0.0000; time*steroid: F(23,644) = 5.9374, *p* = 0.00000).

All females produced more carbon dioxide (V.CO2) during the night compared to the day. EB-treated females produced more V.CO2 during the day and night compared to oil-treated females. Furthermore, EB-treated controls produced more V.CO2 compared to EB-treated Kiss1-GHSR KOs during the night (Figure 3G; steroid: F(1,54) = 131.76, *p* = 0.00000; genotype: F(1,54) = 11.051, *p* = 0.00160; time: F(1,54) = 89.700, *p* = 0.00000; time*steroid: F(1,54) = 15.595, *p* = 0.00023). Like V.O2 consumption, hourly V.CO2 production in EB-treated control females was elevated at 03:00 am, 07:00 am, 12:00 pm, 09:00 pm, 10:00 pm, and 11:00 pm. Further, oil-treated controls had an elevated V.CO2 production compared to oil-treated Kiss1-GHSR KOs at 08:00 pm (Figure 3H; steroid: F(1,27) = 78.343, *p* = 0.00000; genotype: F(1,27) = 6.5286, *p* = 0.01656; time: F(23,621) = 41.501, *p* = 0.0000); time*steroid: F(23,621) = 7.3378, *p* = 0.0000).

Respiratory exchange ratio (RER), a measure of substrate utilization (fat vs. carbohydrates) was higher in EB-treated females at night compared to the day (Figure 3I; steroid: F(1,51) = 5.0672, *p* = 0.02872; time F(1,51) = 27.088, *p* = 0.00000). Hourly RER was increased in EB-treated Kiss1-GHSR KO females compared to oil-treated Kiss1-GHSR KOs at 12:00 am and 07:00 pm, while oil-treated controls revealed a reduced RER compared to EB-treated controls at 02:00 am (Figure 3J; time: F(23,391) = 9.1637, *p* = 0.0000). Higher RER values closer to 1.0 indicate a switch to carbohydrate utilization.

All females exhibited elevated heat production, a measure of energy expenditure, at night compared to the day. Additionally, EB-treated females exhibited an increase in nighttime energy expenditure compared to oil-treated females (Figure 3K; steroid: F(1,54) = 38.796, *p* = 0.00000; genotype: F(1,54) = 5.9726, *p* = 0.01783; time: F(1,54) = 68.971, *p* = 0.00000; time*steroid: F(1,54) = 7.5442, *p* = 0.00816). Hourly energy expenditure was elevated in EB-treated controls at 10:00 pm and 11:00 pm and oil-treated Kiss1-GHSR KO females exhibited reduced energy expenditure compared to oil-treated controls at 06:00 am (Figure 3L; steroid: F(1,27) = 24.597, *p* = 0.00003; time: F(23,621) = 38.603, *p* = 0.0000; time*steroid: F(23,621) = 5.5747, *p* = 0.00000). Collectively, the metabolic, locomotor, and feeding data from the CLAMS support, in part, our hypothesis that GHSR upregulation by E2 in Kiss1 neurons in the ARC of female mice modulates the control of metabolism by the hypothalamus.

### 3.4. Experiment #3: Response to Diet Induced Obesity (DIO)

#### 3.4.1. Body Weight Gain and Body Composition

**Figure 4:**
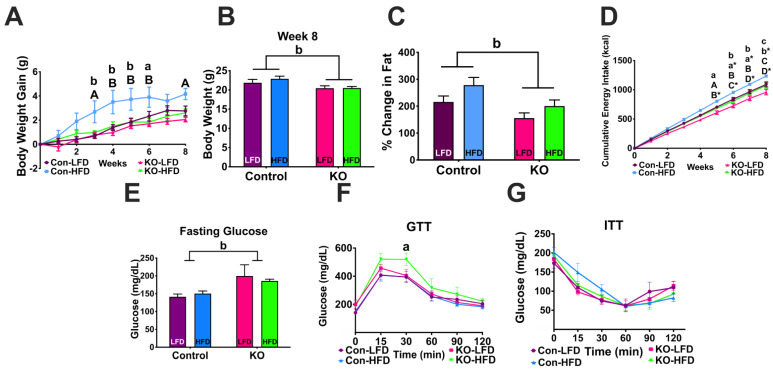
Response to HFD for intact control and Kiss1-GHSR KO (KO) female mice fed LFD or HFD for 8 weeks. (**A**) Weekly body weight gain and (**B**) Week 8 body weight. (**C**) Percent change in fat mass and (**D**) Weekly cumulative energy intake (kCal). (**E**) Fasting (5 h) glucose levels in whole blood, (**F**) Glucose tolerance test (GTT) and (**G**) Insulin tolerance test (ITT). For (**A**,**D**): lowercase letters denote an effect of diet for controls only, lowercase letters with * denote an effect of diet for Kiss1-GHSR-KOs only, uppercase letters denote an effect of genotype on LFD and uppercase letters with * denote an effect of genotype on HFD only. For (**B**,**C**,**E**): Lowercase letters over the lines denote a main effect of genotype. For (**F**): Lowercase letters denote a genotype effect on HFD. A/a = *p* < 0.05; B/b = *p* < 0.01; C/c = *p* < 0.001; D/d = *p* < 0.0001. Data are represented as mean ± SEM and n = 8 mice per group. Data were analyzed with two-way ANOVA with post hoc Holm–Sidak’s multiple comparison test. Mice were 10 weeks of age when placed on LFD or HFD and were 25–30 weeks of age at time of sacrifice.

Females lacking *Ghsr* in Kiss1 neurons are resistant to body weight gain on HFD, as opposed to control mice on HFD (Figure 4A; genotype: F(1,252) = 36.19, *p* < 0.0001; diet: F(1,252) = 35.24, *p* < 0.0001; time: F(8,252) = 24.92, *p* < 0.0001; interaction of genotype*diet: F(1,252) = 10.97, *p* = 0.0011). At the end of the 8-week diet, we observed a main effect of genotype, in that Kiss1-GHSR KO females weighed less than controls, regardless of diet (Figure 4B; genotype: F(1,28) = 8.167, *p* = 0.0080). Furthermore, after 8 weeks of diet, control females had a higher percent change in body fat (Figure 4C; genotype: F(1,28) = 8.775, *p* = 0.0062; diet: F(1,28) = 5.299, *p* = 0.0290), while there were no genotype differences in the change in percent of lean mass, only diet (data not shown; diet: F(1,28) = 4.441, *p* = 0.0442). During weeks 5–8, control females on LFD had greater cumulative energy intake (kCal) compared to controls on HFD, and controls on both diets had a greater energy intake compared to Kiss1-GHSR KO females. In addition, during weeks 6–8 Kiss1-GHSR KO females on LFD had greater cumulative energy intake compared to Kiss1-GHSR KO females on HFD (Figure 4D; kCal; genotype: F(1,108) = 110.9, *p* < 0.0001; diet: F(1,108) = 75.09, *p* < 0.0001; time: F(8,108) = 999.0, *p* < 0.000; time*genotype: F(8,108) = 4.974, *p* < 0.0001; time*diet: F(8,108) = 3.236, *p* = 0.0024). Serum concentration for ghrelin and insulin were measured in control and KO females fed on LFD or HFD. We found ghrelin was elevated in KO females compared to controls on both diets and insulin was reduced in KO females on a HFD compared to control females on a HFD (Figure S9C,D). Overall, deletion of *Ghsr* in Kiss1 neurons reduced cumulative energy intake and abrogated body weight gain on HFD, indicating a crucial role in regulating feeding behavior and subsequent adiposity.

#### 3.4.2. Glucose and Insulin Tolerance Tests

To determine if *Ghsr* expression in Kiss1 neurons is involved in the effect of DIO on glucose homeostasis, we conducted glucose and insulin tolerance tests on all mice. Like our previous experiment, Kiss1-GHSR KO females had an elevated fasting glucose in response to a 5 h fast compared to controls, regardless of diet (Figure 4E; genotype: F(1,28) = 7.806, *p* = 0.0093). In response to a glucose challenge, we observed a decrease in glucose clearance only in Kiss1-GHSR KO females fed HFD, with glucose levels peaking around minute 30, rather than at 15 min for the other groups (Figure 4F; time: F(5,140) = 102.03, *p* = 0.0000). We observed no differences in ITT (Figure 4G). Additionally, there were no differences in the AUC analysis of GTT or ITT (data not shown). This data is similar to our previous findings in that *Ghsr* expression in Kiss1 neurons participates in the regulation of glucose homeostasis.

#### 3.4.3. Locomotor Activity and Ingestive Behaviors in CLAMS

**Figure 5:**
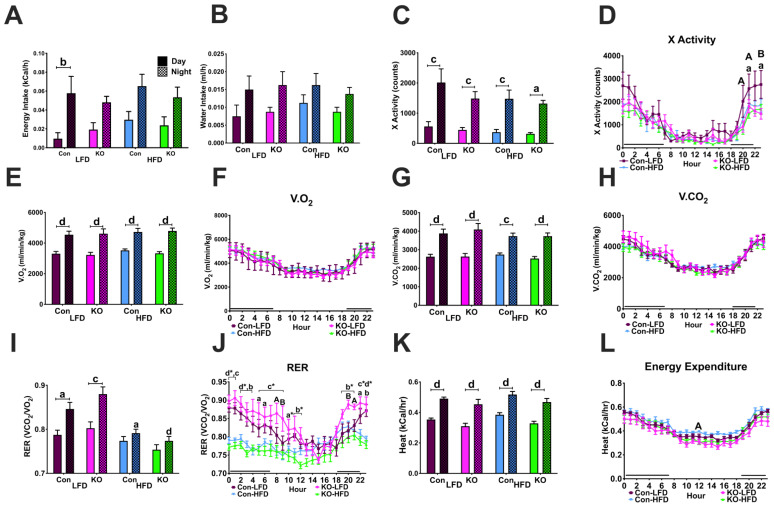
Metabolic response to HFD in intact control and Kiss1-GHSR KO (KO) female mice fed LFD or HFD for 8 weeks. (**A**) Daytime and nighttime energy (kCal) and (**B**) water intake, (**C**) total X-activity (counts) and (**D**) Hourly X-activity. (**E**) Daytime vs. nighttime volume of oxygen consumption (V.O2, ml/min/kg), and (**F**) Hourly volume of oxygen consumption (V.O2, ml/min/kg). (**G**) Daytime vs. nighttime volume of carbon dioxide production (V.CO2, ml/min/kg), and (**H**) Hourly volume of carbon dioxide production (V.CO2, ml/min/kg). (**I**) Daytime vs. nighttime respiratory exchange ratio (RER, V.CO2/V.O2), and (**J**) Hourly respiratory exchange ratio (RER, V.CO2/V.O2). (**K**) Daytime vs. nighttime energy expenditure (Heat, kCal/hr), and (**L**) Hourly energy expenditure (Heat, kCal/hr). For (**D**,**F**,**J**,**H**,**L**): Lowercase letters denote significance between diet in controls, lowercase letters with * denote significance between diet in Kiss1-GHSR-KOs, uppercase letters denote significance between genotypes on LFD. Lowercase letters above capped lines denote significance between time periods within treatment and genotype. A/a = *p* < 0.05; B/b = *p* < 0.01; C/c = *p* < 0.001; D/d = *p* < 0.0001. Data are represented as mean ± SEM and n = 8 mice per group. Data were analyzed with two-way ANOVA with post hoc Holm–Sidak’s multiple comparison test. Mice were 17–19 weeks of age during metabolic experiments.

Control females fed LFD had greater energy intake (kCal) at night compared to the day (Figure 5A; time: F(1,56) = 23.090, *p* = 0.00001). Furthermore, there were no differences in water intake between females (Figure 5B; time: F(1,56) = 10.370, *p* = 0.00213). X-activity was elevated in all females at night compared to the day (Figure 5C; time: F(1,56) = 54.127, *p* = 0.00000). Hourly X-activity was elevated in LFD-fed controls at 08:00 pm, 10:00 pm, and 11:00 pm compared to Kiss1-GHSR KOs. Furthermore, controls on LFD had more X-activity at 10:00 pm and 11:00 pm (Figure 5D; time: F(23,644) = 35.080, *p* = 0.0000; time*genotype: F(23,644) = 1.7458, *p* = 0.01711). This data shows that Kiss1-GHSR KO females, regardless of diet, shared movement patterns with HFD-fed controls, while LFD-fed controls moved more.

#### 3.4.4. Metabolic Parameters in the CLAMS

All females consumed more oxygen (V.O2) during the night compared to the day (Figure 5E; time: F(1,56) = 89.747, *p* = 0.00000) while there were no hourly differences in V.O2 (Figure 5F). All females also produced more carbon dioxide (V.CO2) during the night compared to the day (Figure 5G; time: F(1,56) = 86.938, *p* = 0.00000) while there were no hourly differences in V.CO2 (Figure 5H). RER was higher in LFD-fed females at night compared to the day. In addition, LFD-fed females exhibited an elevated RER compared to HFD fed females of the same genotype at night (Figure 5I; diet: F(1,56) = 41.512, *p* = 0.00000; time: F(1,56) = 25.032, *p* = 0.00001; diet*genotype: F(1,56) = 6.1682, *p* = 0.01603; diet*time: F(1,56) = 8.0857, *p* = 0.00622). Hourly RER revealed that females on LFD had elevated RER, particularly during the night and morning hours, compared to HFD fed mice. Kiss1-GHSR KO females on LFD had elevated RER compared to controls on LFD at 08:00 am, 09:00 am, 08:00 pm and 09:00 pm (Figure 5J; diet: F(3,28) = 9.988, *p* = 0.0001; time: F(23,644) = 17.47, *p* < 0.0001; diet*time: F(69,644) = 3.095, *p* < 0.0001). RER hourly data has been broken up by diet for easier visualization in Figure S5. All females exhibited elevated heat production, a measure of energy expenditure, at night compared to the day (Figure 5K; genotype: F(1,56) = 11.992, *p* = 0.00103; time: F(1,56) = 106.01, *p* = 0.00000). Hourly energy expenditure revealed controls on HFD produced more heat compared to Kiss1-GHSR KOs on HFD at 12:00 pm. These data indicate that *Ghsr* expression in Kiss1 neurons plays a critical role in the metabolic compensation to HFD (Figure 5L; diet: F(3,28) = 3.190, *p* = 0.0389; time: F(23,644) = 74.26, *p* < 0.0001).

### 3.5. Experiment #4: Locomotor and Anxiety-like Behavior

#### 3.5.1. Open Field Test

**Figure 6:**
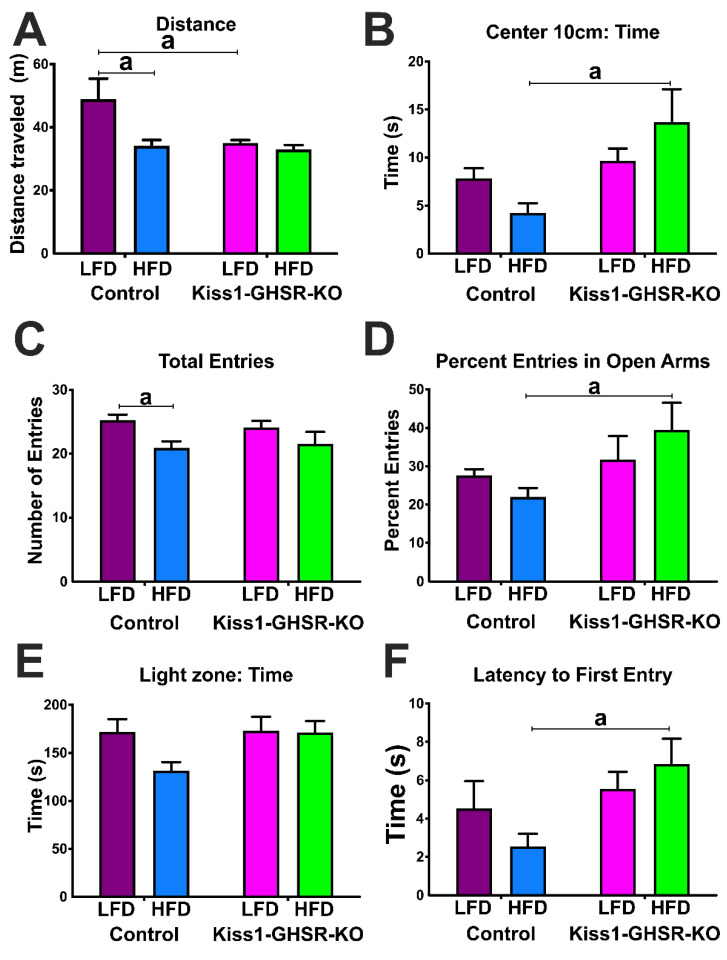
Locomotor and Anxiety-like behaviors in intact control and Kiss1-GHSR KO (KO) female mice fed LFD or HFD for 8 weeks. (**A**) Distance traveled (m) in an Open Field Test (OFT) and (**B**) Time (s) spend in the center zone of OFT. Elevated Plus Maze (EPM) (**C**) Total entries to open arms and (**D**) Percent entries in open arms. Light Dark Box (LDB) (**E**) Time (s) in light zone and (**F**) Latency (s) of first entry to the dark zone (a = *p* < 0.05). Data are represented as mean ± SEM and *n* = 8 mice per group. Data were analyzed with two-way ANOVA with post hoc Holm–Sidak’s multiple comparison test. Mice were 20–25 weeks of age during behavior experiments.

The OFT was utilized to assess exploratory behavior. The evaluation of distance traveled (m) (Figure 6A) revealed controls on LFD traveled further than both Kiss1-GHSR KOs on LFD and controls on HFD (genotype: F(1,41) = 4.151, *p* = 0.0481; diet: F(1,41) = 5.180, *p* = 0.0281). The loss of *Ghsr* in *Kiss1* neurons eliminate the diet-induced reduction in locomotion. On time spent in the center zone (s; 10 cm), we found that Kiss1-GHSR KOs on HFD spent more time in the center zone compared to controls on HFD (Figure 6B; genotype: F(1,41) = 8.375, *p* = 0.0061).

#### 3.5.2. Elevated Plus Maze

The EPM was also used as a measure of avoidance behavior. The assessment of total open arm entries revealed that HFD reduced the number of entries in controls, but not Kiss1-GHSR KOs (Figure 6C; diet: F(1,42) = 7.775, *p* = 0.0079). Furthermore, Kiss1-GHSR KO females on HFD had a significantly higher percentage of entries to open arms compared to controls on HFD (Figure 6D; genotype: F(1,42) = 4.945, *p* = 0.0316).

#### 3.5.3. Light Dark Box

We utilized the LDB in conjunction with the OFT and EPM to assess exploratory and avoidance behavior. In the assessment to time spent in the light zone (s; Figure 6E), we did not observe an effect of diet or genotype. The evaluation of latency to first entry to light zone (s) showed Kiss1-GHSR KOs on HFD took longer to enter the light zone compared to controls on HFD (Figure 6F; genotype: F(1,41) = 5.760, *p* = 0.0210). The combined results from the OFT, EPM, and LDB reveal that *Ghsr* expression in *Kiss1*-expressing neurons plays a role in regulating avoidance behavior on HFD.

### 3.6. Experiment #5: Fasting-Induced Refeeding

**Figure 7:**
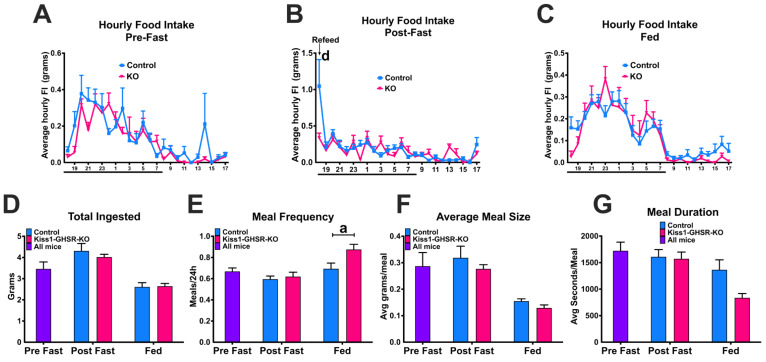
Meal pattern analysis intact control and Kiss1-GHSR KO (KO) female mice on normal chow diet. (**A**) Average hourly food intake for control and KO females 24 h before fasting. (**B**) Average hourly food intake for control and KO females 24 h after fasting. (**C**) Average hourly food intake for control and KO females 24 h of non-restricted feeding. (**D**) Total food ingested over 24 h before fasting, 24 h after fasting, and fed normally. (**E**) Meal Frequency Average 24 h before fasting, 24 h after fasting, and fed normally. (**F**) Average meal size (grams) 24 h before fasting, 24 h after fasting, and fed normally. (**G**) Average meal duration 24 h before fasting, 24 h after fasting, and fed normally. For Lowercase letters denote significance between control and KO females. Lowercase letters above capped lines denote significance between genotypes withing the same treatment (a = *p* < 0.05; b = *p* < 0.01; c = *p* < 0.001; d = *p* < 0.0001). Data are represented as mean ± SEM and n = 14 controls and n = 8 Kiss1-GHSR KO mice per group. Data were analyzed with two-way ANOVA with post hoc Holm–Sidak’s multiple comparison test. Mice were 8–10 weeks of age during BioDAQ experiments.

Prior to fasting and when fed normally, control and Kiss1-GHSR KO females show no hourly differences in food intake (Figure 7A,C). During the first hour of refeeding, however, Kiss1-GHSR KO females did not exhibit an increase in food intake to the same extent as the controls (Figure 7B). Across the 24 h before fasting, 24 h after fasting, and 24 h of ad libitum feeding, total food ingested, meal size, and meal duration was not different between groups (Figure 7D,F,G). Meal frequency was elevated in fed Kiss1-GHSR KO females compared to controls (Figure 7E). Overall, fasting did not have the same effect in females lacking *Ghsr* expression in *Kiss1* neurons as it did with controls.

### 3.7. Experiment #6: Thermoregulation

**Figure 8:**
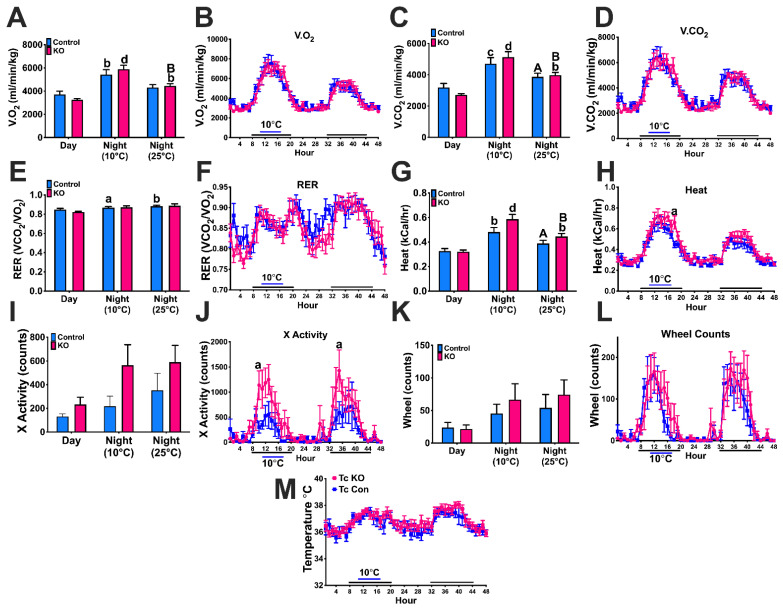
Response to cold stress in intact and chow fed control and Kiss1-GHSR KO (KO) female mice during 6 h overnight cold stress (10 °C). (**A**) Daytime vs. nighttime volume of oxygen consumption (V.O2, ml/min/kg) and (**B**) Hourly volume of oxygen consumption (V.O2, ml/min/kg). (**C**) Daytime vs. nighttime volume of carbon dioxide production (V.CO2, ml/min/kg) and (**D**) Hourly volume of carbon dioxide production (V.CO2, ml/min/kg). (**E**) Daytime vs. nighttime respiratory exchange ratio (RER, V.CO2/V.O2), and (**F**) Hourly respiratory exchange ratio (RER, V.CO2/V.O2). (**G**) Daytime vs. nighttime energy expenditure (Heat, kCal/hr), and (**H**) Hourly energy expenditure (Heat, kCal/hr). (**I**) Daytime and nighttime total X-activity (counts), and (**J**) Hourly X-activity. (**K**) Daytime and nighttime total wheel activity (counts;), and (**L**) Hourly wheel activity (counts). (**M**) Core body temperature (Tc). For (**A**,**C**,**E**,**G**): Lowercase letters denote significance within genotype compared to the day, uppercase letters denote significance within genotype, between night at 10 °C and 25 °C. For (**H**,**J**): Lowercase letters denote significance between genotypes. (A/a = *p* < 0.05; B/b = *p* < 0.01; C/c = *p* < 0.001; D/d = *p* < 0.0001). Data are represented as mean ± SEM and n = 6 mice per group. Data were analyzed with two-way ANOVA with post hoc Holm–Sidak’s multiple comparison test. Mice were 10–12 weeks of age during thermoregulation experiments.

Kiss1-GHSR KO females consumed more oxygen (V.O2) during the night at both 10 and 25 °C compared to the day. In addition, Kiss1-GHSR KO females consumed more V.O2 at 10 °C compared to 25 °C at night, while control females only consumed more V.O2 at 10 °C at night compared to the day (Figure 8A; time: F(2,30) = 29.932, *p* = 0.00000). Hourly V.O2 consumption was no different between groups (Figure 8B; time: F(47,470) = 29.534, *p* = 0.0000). Kiss1-GHSR KO females produced more carbon dioxide (V.CO2) during the night at both 10 and 25 °C compared to the day. Furthermore, Kiss1-GHSR KO females produced more V.CO2 at 10 °C compared to 25 °C at night, while control females only produced more V.CO2 at 10 °C at night compared to 25 °C at night (Figure 8C; time: F(2,30) = 27.231, *p* = 0.00000). Like V.O2 consumption, hourly V.CO2 production consumption was no different between groups (Figure 8D; time: F(47,470) = 28.245, *p* = 0.0000).

RER was closer to 1.0 for all females, indicating the utilization of carbohydrates instead of fat for energy. Only control females exhibited an increase in RER between day and both nights at 10 and 25 °C (Figure 8E; time: F(2,30) = 27.231, *p* = 0.00000). Hourly RER was not different between groups (Figure 8F; time: F(2,30) = 27.231, *p* = 0.00000). All Kiss1-GHSR KO females exhibited elevated heat production, a measure of energy expenditure, both nights at 10 and 25 °C compared to the day. Furthermore, both control and Kiss1-GHSR KO females produced more heat during the night at 10 °C compared to 25 °C (Figure 8G; genotype: F(1,30) = 5.2124, *p* = 0.02968; time: F(2,30) = 28.229, *p* = 0.00000). Hourly energy expenditure was elevated in Kiss1-GHSR KO females compared to controls one hour before lights on (Figure 8H; time: F(47,470) = 28.701, *p* = 0.0000).

X-activity was not significantly different between controls and Kiss1-GHSR KOs; however, there was a main effect of genotype in that Kiss1-GHSR KO females moved more than controls (Figure 8I; genotype: F(1,30) = 5.7932, *p* = 0.02245; time: F(2,30) = 3.2916, *p* = 0.05100). Hourly X-activity was elevated Kiss1-GHSR KO females compared to controls 3–4 h into the dark period (Figure 8J; time: F(47,470) = 7.8575, *p* = 0.0000; time*genotype (trending): F(47,470) = 1.3658, *p* = 0.05939). Furthermore, wheel count activity and hourly core body temperature (Tc) were not significantly different between females (Figure 8K; time: F(2,27) = 3.3772, *p* = 0.04909, Figure 8L; time: F(47,376) = 11.376, *p* = 0.0000 and Figure 8M). These data, taken together reveal the importance of *Ghsr* expression in Kiss1 neurons regarding thermoregulation. Kiss1-GHSR KO females experience elevated metabolic activity and ambulatory activity to maintain the core body temperature during cold stress, indicating a dysregulation in thermoregulation.

### 3.8. Extra-Arcuate Gene Expression

To determine if the deletion of GHSR in Kiss1 neurons altered the expression of *Ghsr* or *Kiss1* in other brain regions where both genes are expressed, we explored *Kiss1* and *Ghsr* gene expression in OVX EB- or Oil- treated females in the BNST and AMG (Figure S6). In addition, we explored intact control and KO females *Kiss1* expression in AVPV, BNST and AMG and *Ghsr* expression in AVPV, BNST, AMG, VTA, and SubN (Figure S8). *Kiss1* was detected in both control and Kiss1-GHSR KO females in all regions without any genotype differences. *Ghsr* expression was reduced in Kiss1-GHSR KO females compared to controls in the BNST and AMG, but no reduction in *Ghsr* was found in the AVPV, substantia nigra, or VTA (Figure S8). These findings indicate the potential for *Kiss1* and *Ghsr* co-expression in regions outside of the hypothalamus with potential influences on mood, motivation, and reward, which currently remain unexplored. Additional experiments would be necessary to make that determination.

### 3.9. Liver Gene Expression

Because Kiss1-GHSR KO females exhibited a higher fasting glucose compared to controls, we determined if we had inadvertently deleted GHSR from kisspeptin expressing cells in the liver (See Supplemental Methods for Liver RNA Extraction and qPCR). *Ghsr* expression was not detectable in either controls or Kiss1-GHSR KO females (data not shown), these findings are corroborated in multiple publications [57,58]. More recent publications have found that liver *Ghsr* expression does increase to detectable values after a 24 h fast, which we did not do [59,60]. These findings indicate that the differences in glucose levels after a 5 h fast are due, in part, to the central deletion of GHSR from Kiss1 neurons and not a peripheral deletion.

## 4. Discussion

It is important to understand the interplay of the neural circuits and hormonal influences that control reproduction, energy balance, and thermoregulation, especially as reproductive complications can result from dietary imbalances in humans. One such interplay in female rodents is the hormonal influence of ghrelin and E2 in the hypothalamic kisspeptin networks to regulate the energetic demands of reproduction. A recent study from our lab found that both negative (24 h fast and 30% caloric restriction) and positive (diet induced obesity) states of energy balance differentially impact the expression of ARC KNDy neuropeptides and their receptors. We demonstrated that E2 can both augment and oppose the effects of positive or negative energy states on KNDy neuropeptides and receptors [31]. Furthermore, our lab has determined that preovulatory levels of E2 increases ARC *Ghsr* expression, the gene that expresses the ghrelin receptor [31], which we found does not occur in males in response to testosterone (Figure S2). Although we did not rule out the actions of testosterone on *Ghsr* expression in other brain regions like DMH, AVPV, AMG and BNST. The increase in *Ghsr* expression in the ARC in females is due to a 6-fold increase in KNDy neurons (*Tac2*), not NPY neurons [49]. In a follow-up study, we found that ghrelin inhibits the M-current, a KCNQ potassium channel current, in KNDy (*Tac2*-GFP) neurons and that E2 induces greater excitability by ghrelin [32]. To elucidate the importance of the interactions of E2 and ghrelin in ARC *Kiss1* neurons and their control of energy homeostasis, we developed a novel *Kiss1*-specific GHSR knockout.

Oil-treated Kiss1-GHSR KO females exhibited reduced *Kiss1*, *Tac2*, *Pdyn*, *Tac3r*, and *Esr1* when compared to oil-treated controls. Furthermore, EB treatment reduced *Kiss1* and *Tac2* expression in both controls and Kiss1-GHSR KOs. However, we only observed a reduction in *Pdyn*, *Tac3r*, and *Esr1* and an increase in *Ghsr* expression in control females with EB treatment. Thus, we confirmed that the E2-induced increase in ARC *Ghsr* expression is indeed due to the increased gene expression in Kiss1 neurons. Another limitation to our model is that the reduction in KNDy-associated genes may be due to the effect of Kiss1^Cre^ heterozygous expression which reduces Kiss1 expression [61] in the Cre mouse model we used. In ghrelin injected OVX females without EB replacement, the differences we observed between control and KO females in expression of *Kiss1*, *Tac2*, *Pdyn*, and *Esr1* were eliminated. This could indicate that the neuropeptides essential for reproduction and LH pulsatility are regulated by an interaction of ghrelin and E2 and that GHSR provides a critical link between nutritional state and reproduction.

**Figure 9:**
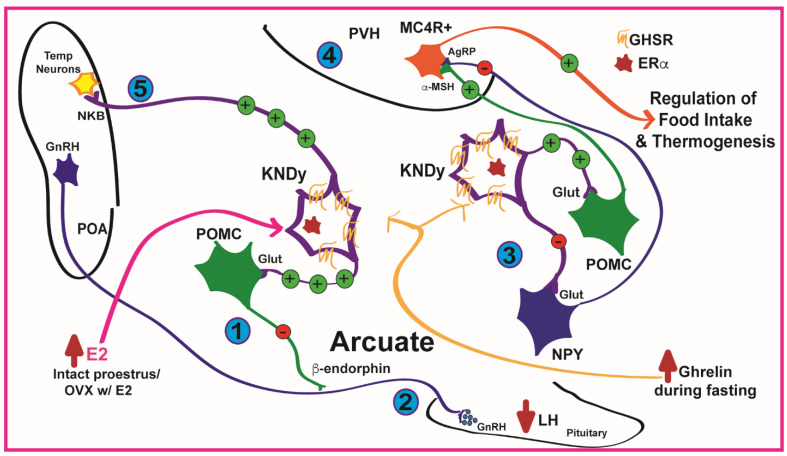
Schematic of hypothesis that E2 increases KNDy sensitivity to ghrelin in females by augmenting Ghsr expression to suppress the LH production during states of elevated ghrelin (caloric restriction), and to diminish the orexigenic input of ghrelin from NPY neurons by activating POMC neurons, via glutamate, and to control thermogenesis and heat dissipation via activation of sympathetic tone from POMC innervation into the PVH and via the release of neurokinin B in the Preoptic Area (POA), respectively. During states of elevated E2 (proestrus or OVX with E2 replacement), Ghsr expression is increased in KNDy neurons augmenting KNDy sensitivity to ghrelin (1). The increase in KNDy sensitivity to ghrelin augments KNDy neuronal output via glutamate onto ARC POMC and NPY/AgRP neurons. Activation of POMC neurons would increase the release of β-endorphin on GnRH neurons thereby suppressing LH (2). The ghrelin increase during fasting enhances KNDy inhibition of NPY/AgRP neurons via glutamate (3) and POMC activation would also increase the release of α-MSH on MC-4 receptor (MC4R)-expressing neurons in the PVH to reduce food intake in opposition to ghrelin’s activation of the feeding drive through NPY neurons (4). Finally, ghrelin-activated KNDy neurons projecting to the POA release NKB to control thermoregulatory output from the POA and heat dissipation (5).

KNDy neurons control energy homeostasis by interacting with the melanocortin circuitry. KNDy neurons directly depolarize POMC neurons through kisspeptin and/or glutamate release and directly or indirectly hyperpolarize NPY/AgRP neurons through glutamate release and/or through an enhancement of inhibitory GABAergic tone [24,25]. Furthermore, POMC and NPY/AgRP neurons modulate KNDy activity [24,26,28,43,44,62], thus producing a neuronal network of three cell types that respond to steroids, nutrients, and peptide hormones to modulate energy homeostasis and reproduction. Based on this information, we conducted a study to examine the importance of E2-induced Ghsr expression in *Kiss1* neurons in the maintenance of energy balance. We hypothesized that E2 increases *Kiss1* neuronal sensitivity to ghrelin in females by augmenting *Ghsr* expression. Greater KNDy activity would enhance the POMC excitability, via glutamate release, while diminishing the orexigenic actions of ghrelin induced NPY neuronal activity. The overall melanocortin tone (more α-MSH release) would increase output from the paraventricular hypothalamus (PVH) and downstream sympathetic tone (see Figure 9).

We found that deleting *Ghsr* in *Kiss1* neurons, regardless of steroid treatment or diet, resulted an elevated blood glucose level in response to a 5 h fast compared to control females. This in conjunction with the lack of *Ghsr* expression in the liver indicates that the result of elevated fasting glucose in Kiss1-GHSR KO females is due to a central effect, not a peripheral artifact. In ghrelin or GHSR null mice, studies reveal they have similar body weight compared to littermates and are not resistant to diet-induced obesity but have been shown that under caloric-restriction there is impaired maintenance of glucose homeostasis [62,63]. POMC and NPY/AgRP neurons play a critical role in the control of glucose homeostasis and hepatic glucose production, particularly in females [64,65]. Glucose tolerance tests performed in POMC knockout female mice found that these mice were glucose intolerant while the same could not be said for males [66]. Ghrelin is a critical mediator of glucose homeostasis [67]. Ghrelin administration increases blood glucose levels and reduces insulin levels, although the opposite effects have been described [68]. The differential effects of ghrelin on glucose homeostasis are dependent on the heteromerization between GHSR and somatostatin receptor 5, both G-protein-coupled receptors (GPCRs) [69]. By deleting *Ghsr* in *Kiss1* neurons, the heteromerization between GHSR and somatostatin receptors in *Kiss1* neurons is potentially disrupted. The lack of heteromerization in addition to decreasing KNDy glutamatergic release, reduces POMC excitability and results in the observed increase in fasting blood glucose. Therefore, activation of the KNDy-POMC circuit by ghrelin in females with high E2 (proestrus) controls hepatic glucose production to ensure sufficient energy is available for elevated locomotor activity and energy expenditure necessary for reproductive behavior.

E2 increases activity levels in female mice; however, in our Kiss1-GHSR KO females, the effect of EB treatment was magnified compared to controls, indicating that *Ghsr* expression in *Kiss1* neurons does play a key role in locomotor activity. We know that POMC and NPY/AgRP neurons project to the ventromedial hypothalamus (VMH) and act through melanocortin-4-receptor (MC4R) to control locomotor behavior. When E2 is high, input to the MC4R in the VMH increases activity in female mice [35]. Thus, during periods of food scarcity (high ghrelin), a female in proestrus (high E2) would have more ghrelin-sensitive Kiss1 neurons, enhancing the activation of POMC neurons and α-MSH release in the VMH [11]. Furthermore, EB-treated KO females exhibited a reduced V.O2 consumption and V.CO2 production compared to EB-treated controls. The modest reduction in metabolism in the KOs is independent of locomotor activity suggesting that the lack of GHSR suppresses sympathetic output directed at metabolic tissues. Collectively, these data indicate that E2’s influence on metabolism, substrate utilization, and energy expenditure is disrupted in Kiss1-GHSR KOs.

We also found that EB-treated controls consumed less than EB-treated Kiss1-GHSR KO females at night, this is a clear indication of the reduced input to POMC neurons in KO females compared to controls. In the HFD study, cumulative food intake was lower in the KO compared to controls in both LFD-fed and HFD-fed mice. Lower HFD intake underlies, in part, the resistance to diet-induced obesity as KOs exhibited reduced body weight and adiposity compared to HFD-fed controls, as previous studies have shown that HFD does not change total caloric intake [70]. While E2-treated KO females consumed more than controls, intact KO females did not respond to a 24 h fast with an increase in food intake as compared to controls. Upon refeeding, controls have a clear surge in food intake immediately upon the return of food, while KO females do not exhibit the same robust spike in food intake, which again is an indication of the importance of ghrelin signaling in *Kiss1* neurons in controlling food intake in females. Furthermore, in exploratory behavioral studies, we observed a reduction in avoidance behaviors in HFD-fed KO females. It is important to note that while body weight can alter avoidance behavior in mice, we saw no differences in locomotor activity in metabolic chambers, between controls and KO females, therefore the differences seen in avoidance behaviors are likely due to the *Ghsr* knockout. These data in conjunction with the reduction of GHSR in both the BNST and amygdala of KO females (Figure S6) indicates that the co-expression of kisspeptin and *Ghsr* in these two regions, along with hypothalamic co-expression, contributes to approach/avoidance behaviors in response to HFD and offers a novel role of kisspeptin neurons in these behaviors [71].

Unlike in the E2-treated OVX females, activity in intact Kiss1-GHSR KO females was not elevated compared to controls and metabolic parameters were no different. Only HFD reduced RER, which indicates the utilization of fat over carbohydrates for energy, a common effect of HFD. Our data is supported by previous findings in which aged GHSR null mice had lower body weight and reduced adiposity; however, they also found elevated metabolic rates (V.O2 and V.CO2), which we did not. As GHSR is only deleted in Kiss1 neurons in our model, our hypothesis is that the differences observed in body weight and adiposity were due to the reduced food intake on HFD due to GHSR deletion in Kiss1 neurons, as activity and metabolic rates were not different. Furthermore, our hypothesis is supported by recent data suggesting a role for POMC and NPY/AgRP neurons in mediating the central control of adiposity by leptin [72]. Leptin receptor activation in both POMC and NPY neurons plays a critical role in the sympathetic innervation of adipose tissue. When leptin receptors are deleted from POMC and NPY neurons, innervation of brown adipose tissue is reduced by 50% and in white adipose tissue is reduced by 30% [72]. While leptin receptor activation in ARC neurons is not the focus of our study, we can postulate that the lack of ghrelin induced Kiss1 activation may interfere with the sympathetic activation by melanocortin neurons leading to changes in adipose sympathetic innervation. Further HFD experiments measuring brown adipose tissue thermogenesis and sympathetic innervation would be necessary to determine if this is true.

Kisspeptin neurons, in conjunction with E2 and ghrelin signaling, have been found to be involved in other homeostatic processes including thermoregulation and activity [26,35]. In mice, which can undergo torpor due to food scarcity and low ambient temperatures, ghrelin administration lowers core body temperature and enhances torpor. Interestingly, the ghrelin-induced torpor response is eliminated by ablation of the ARC [62]. Fasting typically stimulates ghrelin and induces torpor in mice and rats housed in cool environments [73,74]. Our thermoregulation study reveals that deletion of GHSR in Kiss1 neurons modestly increases energy expenditure and induces greater activity in Kiss1-GHSR KO females experiencing a short-term cold stress while maintaining core body temperature. These data indicate that KO females moved more and produced more heat compared to controls to maintain the same core body temperature during cold stress. We hypothesize this is due to ghrelin-induced Kiss1 neuronal activation which results in increased release of neurokinin B in the Preoptic Area to modulate thermoregulation (see Figure 9), as neurokinin B release regulates body temperature in mice [26].

In conclusion, by deleting *Ghsr* expression from ARC Kiss1 neurons, we have revealed a novel central pathway for the control of reproduction and metabolism in female mice. E2 upregulates *Ghsr* expression in ARC *Kiss1* neurons, which, in turn, increases *Kiss1* neuronal sensitivity to ghrelin [28]. This increases input (potentially glutamate) to POMC neurons resulting in an elevated β-endorphin released near GnRH axon terminals in the median eminence to inhibit GnRH release and subsequently LH release from the anterior pituitary, suppressing the HPG axis, until food is available again and ghrelin levels decrease. The activation of the *Kiss1*-melanocortin circuit also reduces food intake and increases activity and metabolism. New insights from our studies show that Kiss1-GHSR KO females exhibited reduced metabolic rates when E2-treated, decreased adiposity due to hypophagia and delayed glucose clearance on HFD, and elevated activity and energy expenditure when cold stressed, we also conclude that the interaction of E2 and ghrelin in *Kiss1* neurons regulates metabolism by altering the activation of the downstream circuits that control sympathetic tone, glucose homeostasis, food intake, and adiposity. This conclusion is supported by our previous findings [28] and by other studies characterizing the Kiss1-POMC-NPY/AgRP circuit [14,22,30,75]. *Ghsr* expression clearly plays a major role in adiposity and glucose homeostasis, without it, these aspects of energy balance will be disrupted. Future studies will investigate both reproductive and metabolic parameters during long-term negative energy balance, i.e., chronic caloric restriction and during times of temperature stress (cold-induced torpor). Collectively, these data suggest that GHSR activation in *Kiss1* neurons modulates metabolism, activity, glucose homeostasis and thermoregulation illustrating one of many novel mechanisms for E2 and ghrelin to control *Kiss1* neurons and their physiological functions.

## Figures and Tables

**Table 1:** List of primers for qPCR.

Gene	Accession Number	Forward Primer	Reverse Primer
*Agrp*	NM_007427.2	CTCCACTGAAGGGCATCAGAA	ATCTAGCACCTCCGCCAAA
*Cart*	NM_013732	GCTCAAGAGTAAACGCATTCC	GTCCCTTCACAAGCACTTCAA
*Esr1*	NM_007956	GCGCAAGTGTTACGAAGTG	TTCGGCCTTCCAAGTCATC
*Gapdh*	NM_008084.2	TGACGTGCCGCCTGGAGAAA	AGTGTAGCCCAAGATGCCCTTCAG
*Ghsr*	NM_177330	CAGGGACCAGAACCACAAAC	AGCCAGGCTCGAAAGACT
*Hprt*	NM_013556	GCTTGCTGGTGAAAAGGACCTCTCGAAG	CCCTGAAGTACTCATTATAGTCAAGGGCAT
*Kiss1*	NM_178260	TGATCTCAATGGCTTCTTGGCAGC	CTCTCTGCATACCGCGATTCCTTT
*Npy*	NM_023456	ACTGACCCTCGCTCTATCTC	TCTCAGGGCTGGATCTCTTG
*Pdyn*	NM_018863	AGCTTGCCTCCTCGTGATG	GGCACTCCAGGGAGCAAAT
*Pomc*	NM_008895	GGAAGATGCCGAGATTCTGC	TCCGTTGCCAGGAAACAC
*Tac2*	NM_001199971	CGTGACATGCACGACTTC	CCAACAGGAGGACCTTAC
*Tac3r*	NM_021382	TACACCATCGTTGGAATTAC	ATGTCACCACCACAATAATC
*Tac2 (Single Cell)*	NM_021382	TCTGGAAGGATTGCTGAAAGTG	GTAGGGAAGGGAGCCAACAG

## Data Availability

Data is available upon request.

## Supplementary Materials

The following supporting information can be downloaded at: https://www.mdpi.com/article/10.3390/biom12101370/s1, Supplemental Methods for Vaginal opening and cytology, fertility, LH blood collection and assay, Cell harvesting of dispersed Tac2-GFP and Kiss1-GFP neurons, Liver RNA extraction and quantitative real-time PCR, and serum insulin and ghrelin measurements. In addition Table S1: Average Cq values for Figure 1 qPCR; Figure S1: GHSR expression in Kiss1 neurons from Control (Tac2-GFP) and Experimental (Kiss1^Cre/+/^GHSR^fl/fl^) female mice; Figure S2: GHSR signaling in Tac2 neurons from GDX male mice is not regulated by TP; Figure S3: LH pulsatility; Figure S4: Arcuate gene expression; Figure S5: Hourly respiratory exchange ratio; Figure S6: OVX control and Kiss1-GHSR KO females with or without E2 replacement; Figure S7: Arcuate melanocortin gene expression in OVX control and Kiss1-GHSR KO females; Figure S8: Gene expression in control and Kiss1-GHSR KO females; Figure S9: Serum concentration of Ghrelin and Insulin in control and Kiss1-GHSR KO females [46,75,76,77,78,79,80,81].
