# Supplementary material for: Deletion of Growth Hormone Secretagogue Receptor in Kisspeptin Neurons in Female Mice Blocks Diet-Induced Obesity
Source: Biomolecules. 2022 Sep 25;12(10):1370. doi: 10.3390/biom12101370 (PMC9599822; doi:10.3390/biom12101370)
Supplement: Supplementary file 1 [file biomolecules-12-01370-s001.zip › biomolecules-1914732-supplementary.pdf]

biomolecules

MDPI

Supplementary Materials

1. Supplemental Methods

1.1. Vaginal Opening and Cytology

To determine if the loss of *Ghsr* in *Kiss1* neurons altered the onset of puberty weaned female pups (PND 21) were visually checked daily for vaginal opening as an indication of pubertal age (*n* = 12). To determine if the loss of *Ghsr* in *Kiss1* neurons altered the estrous cycle, after vaginal opening, vaginal lavage was performed to determine the estrous cycle stage daily for two weeks. We assessed the ratio of nucleated and cornified squamous epithelial cells as well as leukocytes to identify the estrous stage.

1.2. Fertility

To determine if the loss of *Ghsr* in *Kiss1* neurons alters fertility, control females were bred with Kiss1-GHSR KO males and control males with Kiss1-GHSR KO females (*n* = 3 of each genotype). All female breeders were paired on the morning of proestrus. Females were checked for seminal plugs to determine day one of pregnancy. All females remained paired for three subsequent litters, time to parturition, litter size, and sex ratio were recorded.

1.3. LH Blood Collection and Assay

To determine if the loss of *Ghsr* in *Kiss1* neurons alters LH pulsatility, an LH assay was performed using the same protocols as previously published [75,77]. Briefly, all mice were handled daily between 09:30 am–10:30 am for three weeks prior to tail bleed to reduce stress. All LH collections took place from 10:00 am to 01:00 pm. For gonadally intact mice, collections took place in either diestrus or proestrus. A small drop of blood was gently massaged from the tip of the tail and 6 µL was collected then immediately placed in 54 µL of prepared buffer, mixed and placed on dry ice. Samples were stored at −80 °C until shipping for analysis. Buffer was prepared following Center for Research in Reproduction Ligand Assay and Analysis Core guidelines.

The Ultra-Sensitive Mouse & Rat LH ELISA is an in-house method performed by the Center for Research in Reproduction Ligand Assay and Analysis Core based on a manuscript published by Steyn et al. [75]. This assay allows for a LH measurement in 2.5–5 µl of serum, plasma, or whole blood. The capture monoclonal antibody (anti-bovine LH beta subunit, 518B7) is provided by Janet Roser, University of California. The detection polyclonal antibody (rabbit LH antiserum, AFP240580Rb) is provided by the National Hormone and Peptide Program (NHPP). HRP-conjugated polyclonal antibody (goat anti-rabbit) were purchased from DakoCytomation (Glostrup, Denmark; D048701-2). Mouse LH reference prep (AFP5306A; NHPP) is used as the assay standard. The limit of quantitation (functional sensitivity) was defined as the lowest concentration that demonstrates accuracy within 20% of expected values and intra-assay coefficient of variation (%CV) <20% and was determined by serial dilutions of a defined sample pool. Intra-assay %CV is 2.2%. Inter-assay %CVs are 7.3% (Low QC, 0.13 ng/mL), 5.0% (Medium QC, 0.8 ng/mL) and 6.5% (High QC, 2.3 ng/ml). Functional sensitivity of the USLH assay was determined by serial dilution of a mouse LH reference preparation over 5 assays. Functional sensitivity is defined as the lowest LH concentration with an intra-assay coefficient of variation <20% and LH recovery within 20% of expected concentrations. The assay functional sensitivity (i.e., sample in the well) is 0.016 ng/mL. Corrected sensitivity (which takes into account the dilution factor) is 0.160 ng/ml. LH pulse peaks were identified by using previously established criteria [77,78]. A LH pulse peak was defined by the following 3 criteria: 1) the LH peak value must have a >20% increase compared with the previous (1 or 2) LH values; 2) the LH peak value must be followed by a decrease of >10% in the subsequent (1 or 2) LH values, and 3) the change in pulse amplitude was ≥0.32 ng/ml. After identifying pulse

peaks for each female, we identified the total number of LH peaks during the three-hour blood collection, the average LH concentration, the peak amplitude, defined as the difference in LH concentration between a peak its preceding nadir, and the interpulse interval, defined as time between pulses.

1.4. Cell Harvesting of Dispersed Tac2-GFP and Kiss1-GFP Neurons

To determine if *Ghsr* expression is similarly regulated by testosterone in ARC KNDy (*Tac2*) neurons, we repeated previous experiments done in females [31,79]. GDX-*Tac2*-GFP males received either TP (100 µg) or oil-treatment as previously described. *N* = 6 mice per group (10 single cells and 3 pools of 5 per mouse). Testosterone propionate (TP) were purchased from Steraloids (Newport, RI, USA). Adult *Tac2*-EGFP males were gonadectomized (GDX) under isoflurane anesthesia using similar sterile no-touch technique according to the NIH Guidelines for Survival Rodent Surgery. Following GDX, males were separated into two treatment groups: oil and testosterone propionate (TP; 100 µg; suspended in sesame oil).

We collected *Tac2*-GFP neurons to determine cell-type specific changes in gene expression similar to our previous publications [18,79]. Briefly, animals were sedated with ketamine (100 mL of 100 mg/mL stock, IP) and decapitated. Brains were transferred to a vibratome containing cold, oxygenated aCSF and sliced into 250 *µ*M thick basal hypothalamic (BH) slices. BH slices were transferred to an auxiliary chamber (~1 h) containing oxygenated aCSF. The ARC was microdissected and incubated in a papain solution (40 min at 30 °C) and washed with low calcium artificial cerebrospinal fluid (aCSF) followed by regular aCSF. The ARC was triturated using flame-polished glass Pasteur pipettes to disperse cells, which were placed on a glass-bottomed Petri dish (60 mm) and perfused with aCSF for the duration of the experiment (2 mL/min). *Tac2*-EGFP cells were visualized using a Leica DM-IL fluorescent microscope, patched, and harvested by applying low negative pressure to the pipette using the Xenoworks manipulator system (Sutter Instruments, Novato, CA). Positive pressure was used to expel the contents of the pipette into a siliconized microcentrifuge tube containing: 1 *µ*l 5X Superscript III Buffer (Life Technologies), 15 U Rnasin (Promega), 0.5 *µ*l 100 mM DTT, and DEPC-treated water in 8 *µ*l total volume. *Tac2* neurons were harvested both individually as single cells and collected into 3 pools of 5 *Tac2* neurons from each animal.

Harvested single cells and pooled cells were reverse transcribed as previously described [18,46]. One single cell and one tissue RNA tube were used as negative controls, processed without RT. aCSF was collected every pool or 5 single cells to analyze for contamination. *Tac2*$^{+}$ neurons were analyzed using standard PCR protocols and gel electrophoresis as previously described [46,80]. Primers for single cell PCR are the same as those used with ARC tissue qPCR (Table 1), with the exception of Tac2: F: 50 TCTGGAAGGATTGCTGAAAGTG-3′; R: 50-GTAGGGAAGGGAGCCAACAG-3′ [31]. Negative (cell and tissue samples without RT), aCSF, and positive tissue controls were analyzed with each PCR run.

1.5. Liver RNA Extraction and Quantitative Real-Time PCR

To determine if the deletion of GHSR in *Kiss1*-expressing cells altered the expression of *Ghsr* or *Kiss1* in the liver (Experiment #10), we extracted RNA from the liver of 6 controls and 6 Kiss1-GHSR KO females as previously described [47]. For collection of liver samples, mice were decapitated after an injection of ketamine at 1000 h (100 µL of 100 mg/mL, IP) and the liver was removed. Livers were transferred to RNALater (Life Technologies) and stored overnight at -80 °C until extraction. Liver RNA was extracted using a Trizol extraction coupled with a Macherey-Nagel NucleoSpin kit (Bethlehem, PA). To extract the hepatic RNA, ~20 mg of liver was used. RNA quantity and quality were determined using the same methods as ARC, BNST, and CeA [47].

As previously described [47], cDNA was synthesized, and quantitative real-time PCR (qPCR) was conducted using the primers found in Table 1. Four µl of cDNA was amplified by SSO Advanced (BioRad, Hercules, CA) Master Mix using standard protocols. Relative gene expression was determined using the δδCT method calculated by the geomean of reference genes *Hprt* and *Gapdh* [48,76,81]. Efficiencies were calculated as a percent efficiency and were approximately equal (90%– 110% or one doubling per cycle). Positive, negative and water blank controls were included in the qPCR plate design. The geomean of the Cq values from each reference gene was used to calculate relative gene expression [49].

1.6. Serum Insulin and Ghrelin Measurements

Trunk blood was collected in K+-EDTA coated tubes with the addition of proteinase inhibitor 4-(2-aminoethyl) benzenesulfonyl fluoride hydrochloride (1 mg/ml, Sigma-Aldrich) to protect against peptide degradation. Samples were maintained on ice until centrifugation at 1,000× *g* for 15 min at 4 °C. Plasma supernatant was collected and stored at −80 °C until analysis for insulin, and ghrelin levels, using a multiplex assay (MMHMAG-44 K, EMD Millipore, Billerica, MA).

2. Supplemental Data

Table S1. Average Cq Values for Figure 1 qPCR.

Genotype	Treatment	Reference Cq Values	Kiss1	Tac2	Pdyn	Tac3R
Control	Oil	23.44 ± 0.69	24.77 ± 0.75	22.67 ± 0.94	24.22 ± 0.73	27.53 ± 1.42
	Oil + Ghrelin	24.67 ± 0.24	26.37 ± 1.08	24.84 ± 0.67	25.62 ± 0.37	28.64 ± 0.34
	E2	24.42 ± 0.42	30.28 ± 0.24	26.97 ± 1.45	26.71 ± 0.62	30.51 ± 0.91
	E2 + Ghrelin	24.60 ± 0.37	30.84 ± 1.03	26.18 ± 0.92	26.64 ± 0.54	30.84 ± 0.65
Kiss1-GHSR-KO	Oil	23.96 ± 0.71	27.39 ± 1.53	24.63 ± 1.16	25.96 ± 1.14	29.53 ± 1.24
	Oil + Ghrelin	24.50 ± 0.38	26.76 ± 0.54	24.74 ± 0.62	25.92 ± 0.75	29.37 ± 0.84
	E2	24.34 ± 0.44	31.12 ± 0.68	26.62 ± 0.96	26.68 ± 0.76	30.77 ± 0.49
	E2 + Ghrelin	23.68 ± 0.61	30.53 ± 1.06	26.16 ± 0.74	26.08 ± 0.34	30.40 0 ±.53

Genotype	Treatment	Esr1	Ghsr	Pomc	Agrp	Cart	Npy
Control	Oil	25.97 ± 0.42	28.43 ± 0.68	23.48 ± 0.47	27.89 ±1.14	24.08 ± 0.52	24.27 ± 0.96
	Oil + Ghrelin	27.7 ± 0.65	28.94 ± 0.83	25.8 ± 0.49	28.31 ± 0.59	26.48 ± 0.53	24.31 ± 0.60
	E2	28.32 ± 0.52	27.77 ± 0.85	26.34 ± 0.37	27.70 ± 1.26	26.98 ± 0.54	24.81 ± 0.56
	E2 + Ghrelin	28.55 ± 0.57	28.59 ± 0.63	26.16 ± 0.33	26.44 ± 1.19	26.96 ± 0.36	24.36 ± 0.35
Kiss1-GHSR-KO	Oil	27.77 ± 1.08	32.99 ± 3.86	25.94 ± 1.09	29.84 ± 1.44	26.39 ± 1.12	25.41 ± 1.42
	Oil + Ghrelin	27.39 ± 0.49	31.0 ± 1.05	25.33 ± 0.40	27.70 ± 0.39	26.14 ± 0.44	24.42 ± 0.45
	E2	28.11 ± 0.58	29.82 ± 0.91	24.78 ± 0.40	27.62 ± 0.49	26.3 ± 0.33	24.51 ± 0.51
	E2 + Ghrelin	27.35 ± 0.55	29.62 ± 1.77	25.65 ± 0.75	27.43 ± 0.74	26.49 ± 0.67	23.95 ± 0.69

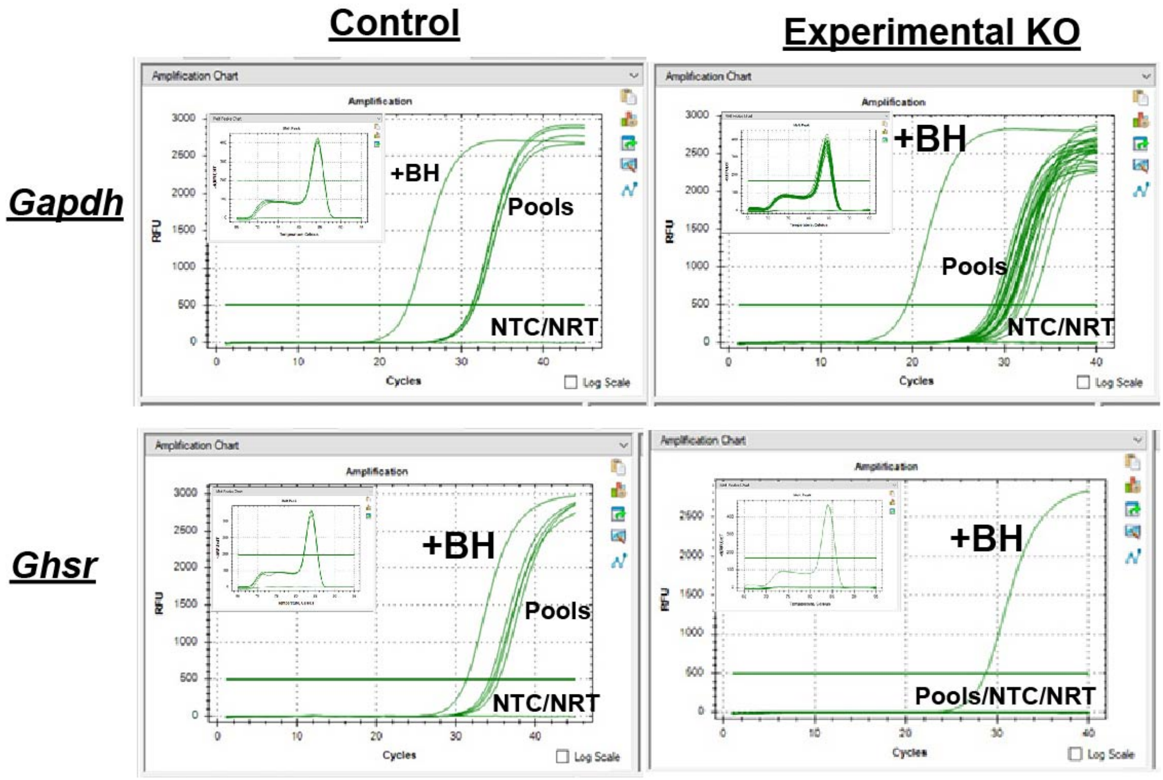

Figure S1. GHSR expression in Kiss1 neurons from Tac2-GFP and Kiss1$^{Cre/+}$/GHSR$^{fl/fl}$ female mice. Cq traces from pools of Kiss1 neurons (Pools) for Gapdh and Ghsr in females that express Ghsr in Kiss1 neurons and those that do not express Ghsr along with positive controls (+BH), no tissue controls (NTC), no reverse transcriptase controls (NRT), and CSF controls. Gapdh expression was used as a reference to indicate proper cell harvesting. Solid horizontal green line is threshold for Cq values. Insets are the melting curves. harvested from female mice. No expression of Ghsr was detected in the Kiss1$^{Cre/+}$/GHSR$^{fl/fl}$ mice as the single trace is the positive control. We used Tac2-GFP mice as without Cre expression, there is no GFP expression in Kiss1$^{+/+}$/GHSR$^{fl/fl}$ for single-cell harvesting.

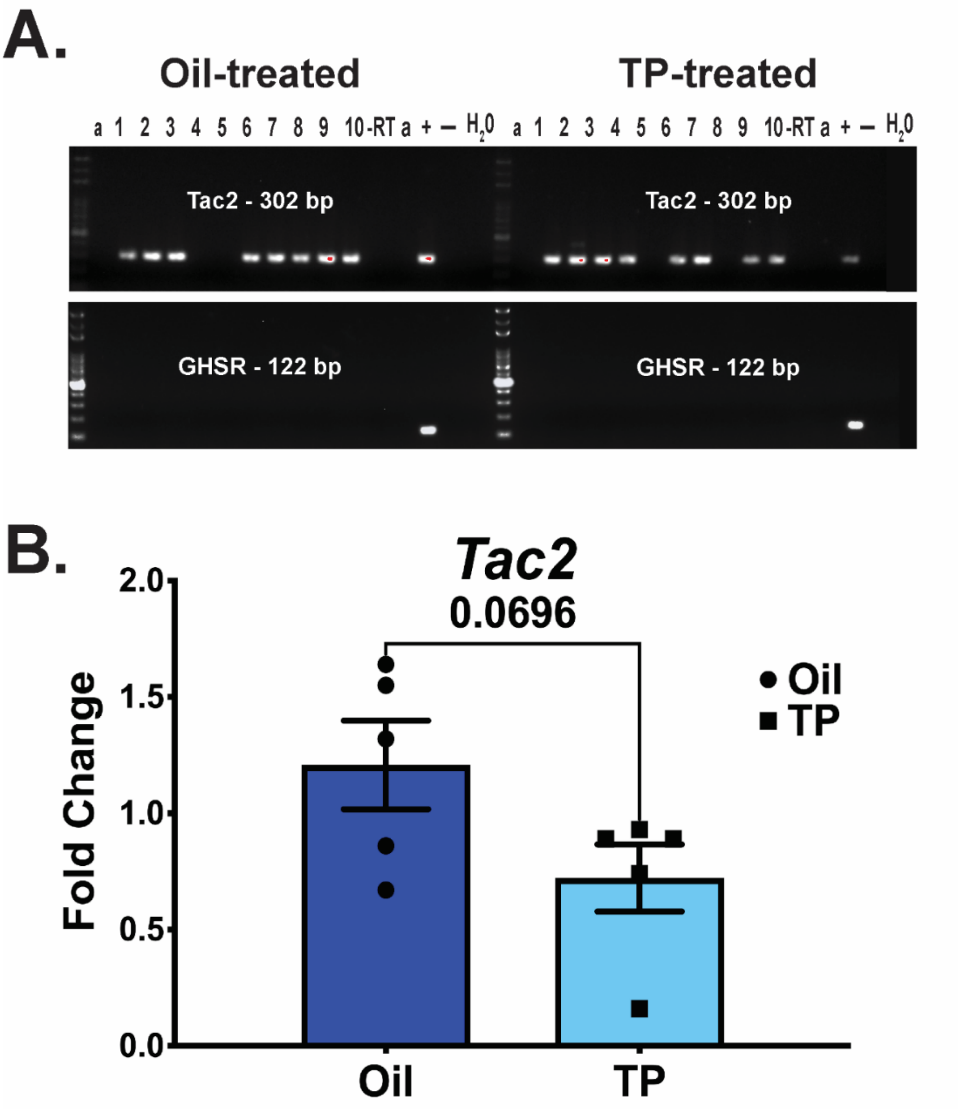

Figure S2. *Ghsr* expression in *Tac2* neurons from GDX male mice is not regulated by TP. (**A**) Representative gel is single-cell PCR amplification products in GDX/oil- and TP-treated male mouse *Tac2* neurons. a = artificial CSF; -RT = without reverse transcriptase (**B**) *Tac2* gene expression in *Tac2*-GFP neuron pools (5 cells per pool). No *Ghsr* expression was detected in *Tac2*-GFP pools. Data were analyzed by Student's t-test.

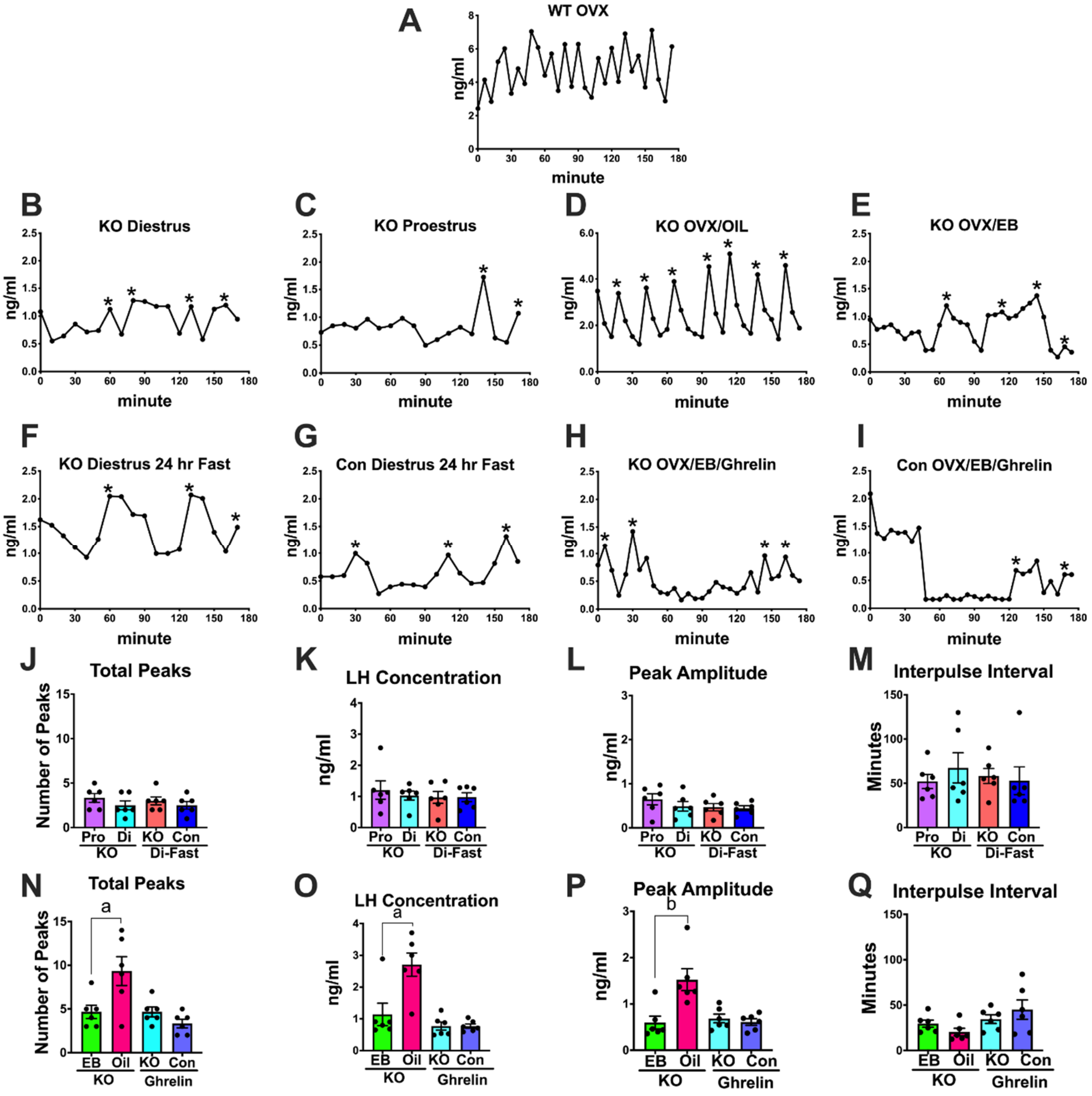

Figure S3. LH pulsatility of: (**A**) OVX WT female for comparison to (**B**) Intact Kiss1-GHSR KO diestrus; (**C**) Intact Kiss1-GHSR KO proestrus; (**D**) Kiss1-GHSR KO OVX/OIL; (**E**) Kiss1-GHSR KO OVX/EB; (**F**) Intact Kiss1-GHSR KO diestrus 24hr fasted; (**G**) Intact control diestrus 24hr fasted; (**H**) Kiss1-GHSR KO OVX/EB/ghrelin (1mg/kg; IP) 30 min prior to collection; (**I**) Control OVX/EB/ghrelin (1 mg/kg; IP) 30 min prior to collection. LH dynamics of OVX females, parameters analyzed were: (J/N) Total LH peaks; (K/O) Average LH concentration; (L/P) Average peak amplitude and (M/Q) Average interpulse interval. [LH] was analyzed at the University of Virginia Ligand Assay and Analysis Core. n = 6 mice per group. Data were analyzed by students t-test. B-I) * indicates LH Peak. J-Q) a = p <0.05, b= p <0.01. Mice were 8–12 weeks of age at time of collection.

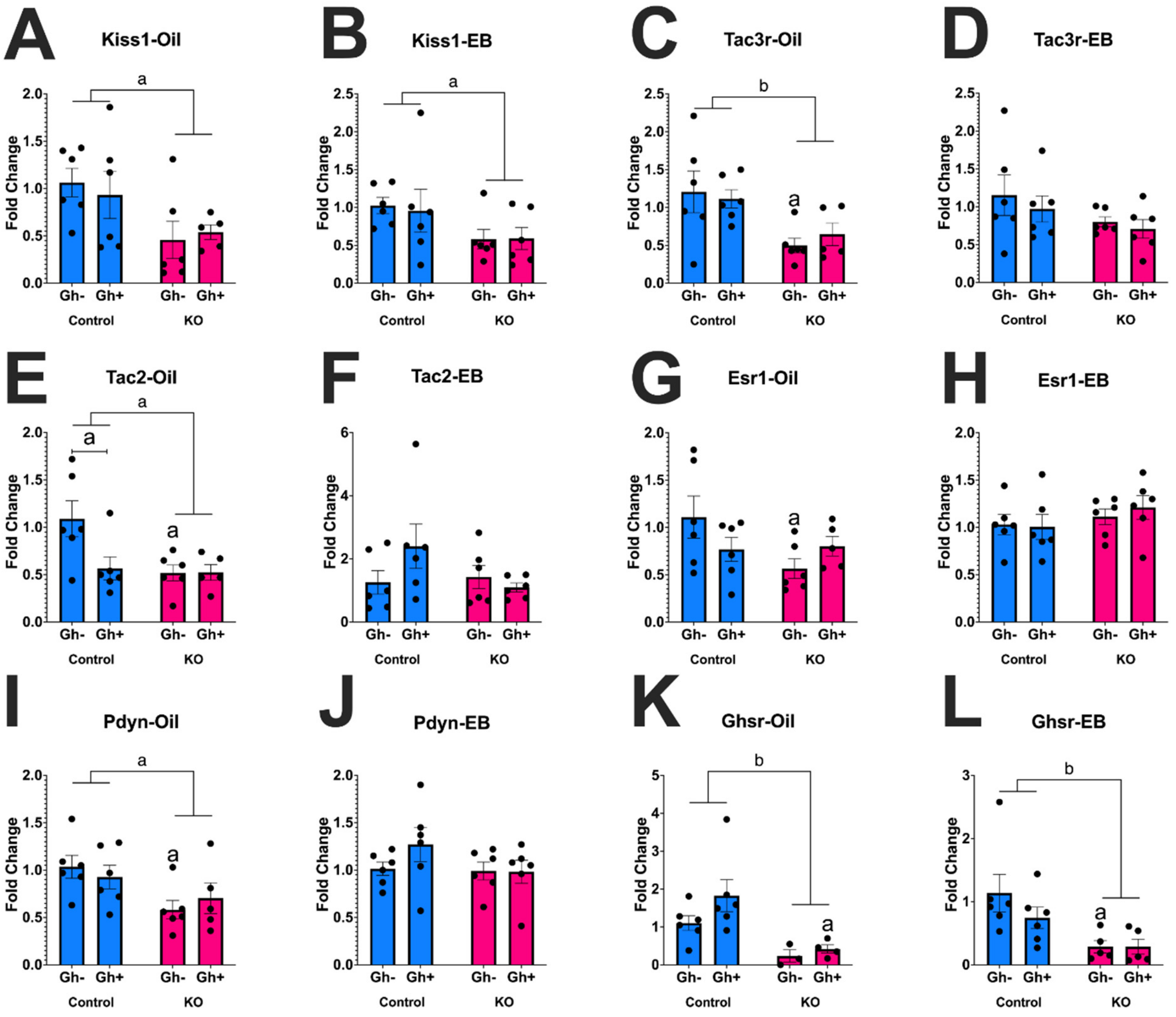

Figure S4. Arcuate gene expression (corresponding to Figure 1) in OVX control and Kiss1-GHSR KO females with or without E2 replacement and with Ghrelin injection: *Kiss1* in oil (**A**) and EB-treated (**B**) females; *Tac3r* in oil (**C**) and EB-treated (**D**) females; *Tac2* in oil (**E**) and EB-treated (**F**) females; *Esr1* in oil (**G**) and EB-treated (**H**) females; *Pdyn* in oil (**I**) and EB-treated (**J**) females; and *Ghsr* in oil (K) and EB-treated (**L**) females. For all graphs, data were analyzed by a two-way ANOVA with post hoc Holm–Sidak's multiple comparison test. Lower case letters above lines denote a main effect of genotype, lowercase letters above capped lines denote a difference between without and with ghrelin (Gh- and GH+, respectively), finally lowercase letters above bars indicate a difference in KO compared to control of the same treatment. (a = $p < 0.05$; b = $p < 0.01$; c = $p < 0.001$; d = $p < 0.0001$). Data is represented as mean ± SEM. Ghrelin injection was given IP at 1 mg/kg 12 h prior to sacrifice.

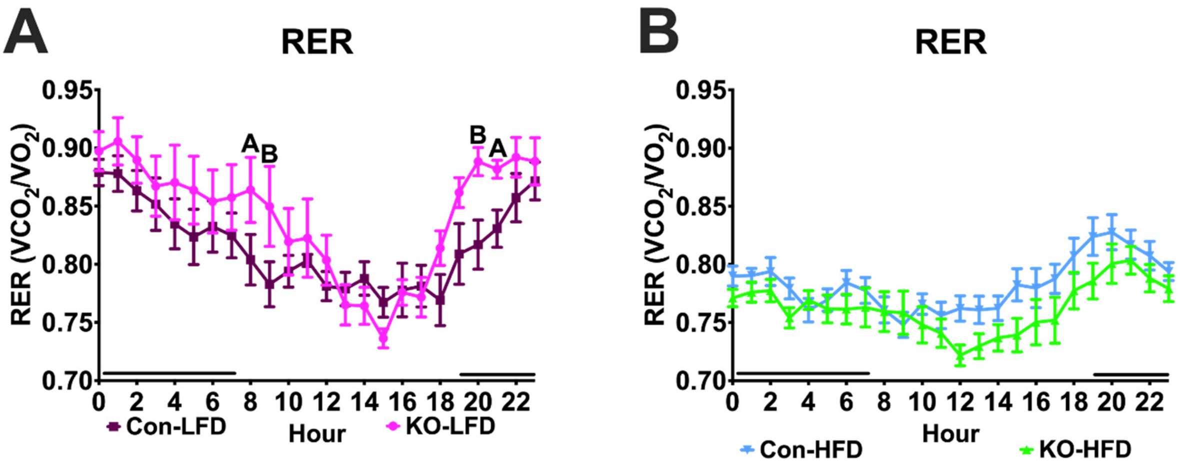

Figure S5. Hourly respiratory exchange ratio (RER; V.CO2/V.O2) for control and KO females from Figure 5J separated as LFD-fed (**A**) or HFD-fed (**B**). Uppercase letters denote significant differences between genotypes. Data are presented as mean ± SEM. A = $p < 0.05$, B = $p < 0.01$.

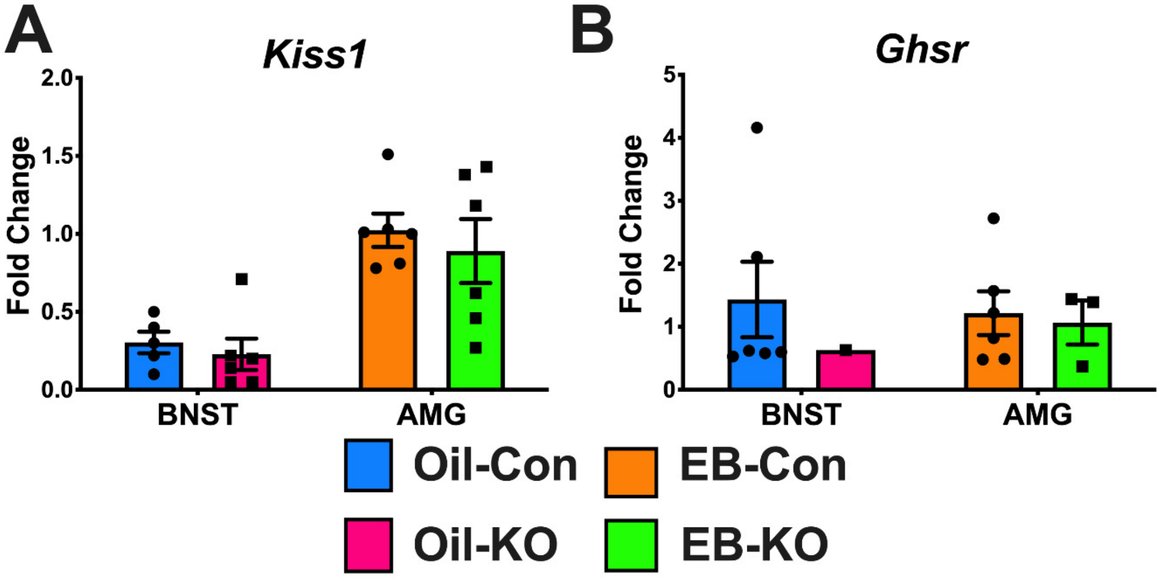

Figure S6. OVX control and Kiss1-GHSR KO females with or without E2 replacement. (**A**) *Kiss1*; (**B**) *Ghsr* expression in BNST and amygdala. For all graphs, data were analyzed by a multi-factorial ANOVA (steroid, genotype) with post hoc Holm–Sidak's multiple comparison test. Data is represented as mean ± SEM.

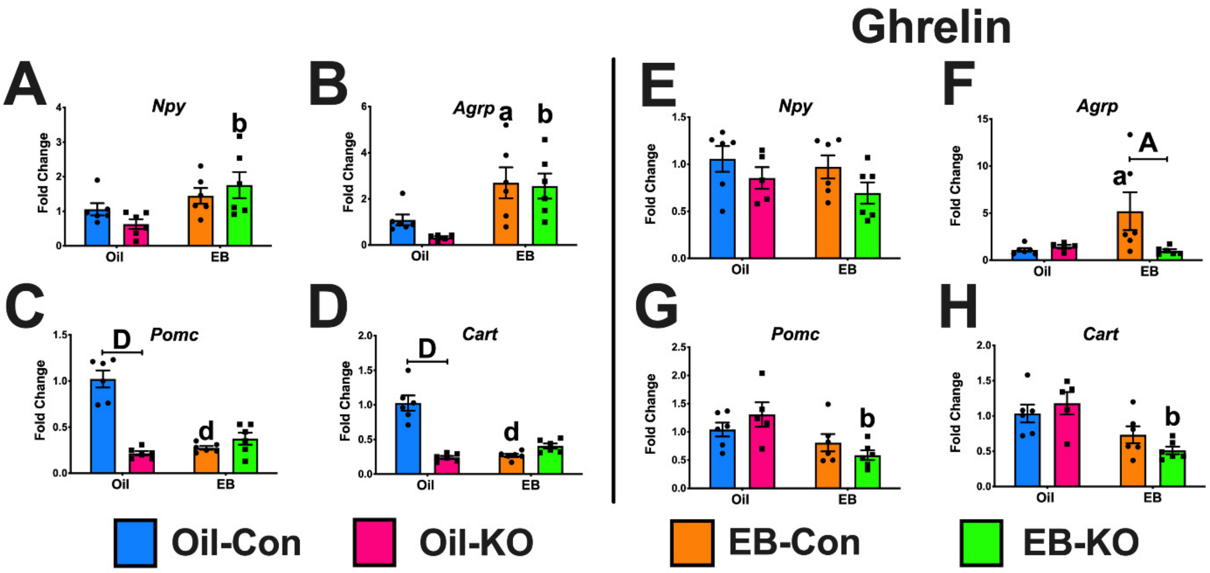

Figure S7. Arcuate melanocortin gene expression in OVX control and Kiss1-GHSR KO females with or without E2 replacement (**A–D**) and with ghrelin injection (E-H): (A/E) Npy; (B/F) Agrp; (C/G) Pomc; and (D/H) Cart. For all graphs, data were analyzed by a multi-factorial ANOVA (steroid, genotype) with post hoc Holm–Sidak's multiple comparison test. Lower case letters denote steroid differences within genotype, uppercase letters above capped lines denote genotype effects (A/a = $p < 0.05$; B/b = $p < 0.01$; C/c = $p < 0.001$; D/d = $p < 0.0001$). Data is represented as mean ± SEM. Ghrelin injection was given IP at 1 mg/kg 12 h prior to sacrifice.

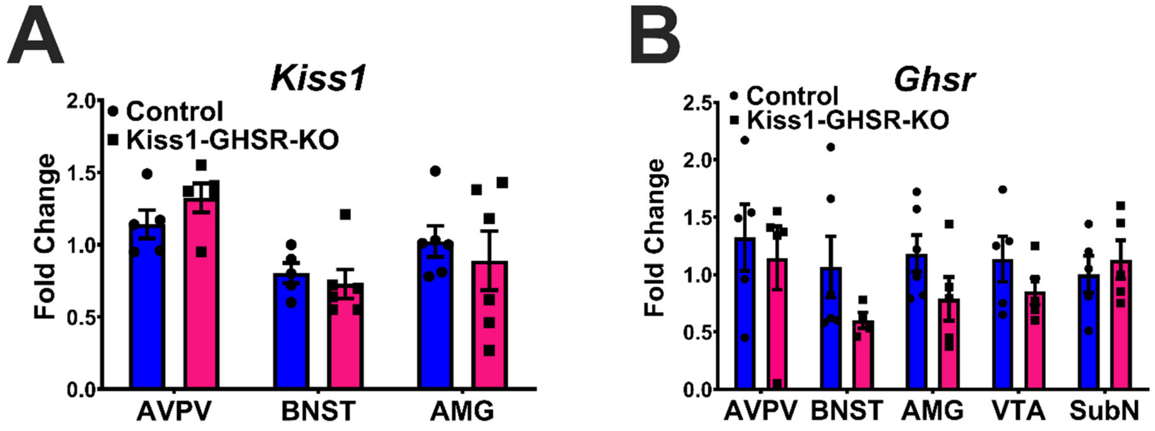

Figure S8. Gene expression in control and Kiss1-GHSR KO females (**A**) *Kiss1* expression in AVPV, BNST, and AMG; (**B**) *Ghsr* expression in AVPV, BNST, AMG, VTA, and SubN. For all graphs, data were analyzed by two-way ANOVA with post hoc Holm–Sidak's multiple comparison test. Data is represented as mean ± SEM.

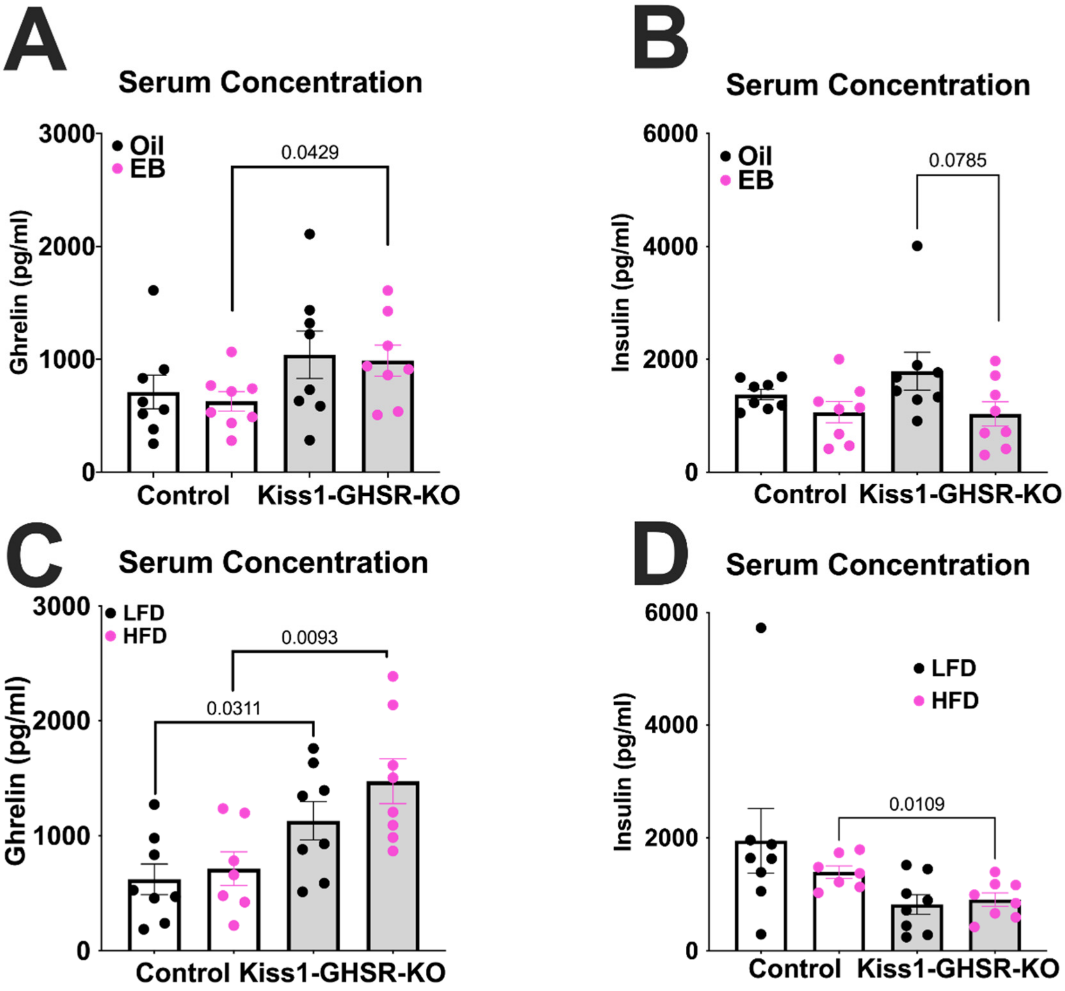

Figure S9. Serum concentration of Ghrelin and Insulin in control and Kiss1-GHSR KO females. Ghrelin (**A**) and Insulin (**B**) concentration in Oil and EB-treated females. Ghrelin (**C**) and (**D**) Insulin concentration in females on LFD or HFD. For all graphs, data were analyzed by two-way ANOVA with post hoc Holm–Sidak's multiple comparison test. Data is represented as mean ± SEM.

2.1. BNST and Central Amygdala Gene Expression

To determine if the deletion of GHSR in *Kiss1* neurons altered the expression of *Ghsr* or *Kiss1* in other brain regions where both genes are expressed, we explored *Kiss1* and *Ghsr* gene expression in the BNST as well as the CeA. Using similar methods as described in Arcuate RNA Extraction and quantitative real-time PCR, *Kiss1* was detected in both control and Kiss1-GHSR KO females in both regions without any genotype differences. These findings indicate the potential for *Kiss1* and *Ghsr* co-expression in regions outside of the hypothalamus with potential influences on mood, motivation, and reward, which currently remain unexplored. Additional experiments would be necessary to make that determination.

2.2. Arcuate Melanocortin Gene Expression

To determine if our selective knockout altered the effects of E2 and ghrelin on melanocortin neuropeptide genes (*Pomc*, *Cart*, *Npy*, *Agrp*), we also measured the expression of these genes in the arcuate samples from Experiment #2. In E2-treated females, *Npy* and *Agrp* were elevated in both genotypes (Figure S7 A & B). Conversely, E2 reduced *Pomc* and *Cart* expression only in control females with a ~75% reduction in both genes in the Kiss1-GHSR KO in oil-treated females (Figure S7 C & D). When ghrelin was administered, there was no effect of genotype or E2 on *Npy* expression (Figure S7 E). *Agrp* expression

was again increased by E2 in control females but not in the Kiss1-GHSR KO. Finally, the genotype effect on *Pomc* and *Cart* expression was eliminated (Figure S7 G & H), with E2 reducing both only in the Kiss1-GHSR KO.

References

18. Bosch, M.A., K.J. Tonsfeldt, and O.K. Rønnekleiv, *mRNA expression of ion channels in GnRH neurons: Subtype-specific regulation by 17β-estradiol.* Molecular and Cellular Endocrinology, 2013. **367**: p. 85-97.
31. Yang, J.A., et al., *The interaction of fasting, caloric restriction, and diet-induced obesity with 17beta-estradiol on the expression of KNDy neuropeptides and their receptors in the female mouse.* Mol Cell Endocrinol, 2016. **437**: p. 35-50.
46. Roepke, T.A., et al., *Fasting and 17β-estradiol differentially modulate the M-current in NPY neurons.* The Journal of neuroscience : the official journal of the Society for Neuroscience, 2011. **31**(33): p. 11825-11835.
47. Adams, S., et al., *Sex- and age-dependent effects of maternal organophosphate flame-retardant exposure on neonatal hypothalamic and hepatic gene expression.* Reprod Toxicol, 2020. **94**: p. 65-74.
48. Krumm, E.A., et al., *Organophosphate Flame-Retardants Alter Adult Mouse Homeostasis and Gene Expression in a Sex-Dependent Manner Potentially Through Interactions With ERα.* Toxicological Sciences, 2017: p. kfx238-kfx238.
49. Yasrebi, A., et al., *Differential gene regulation of GHSR signaling pathway in the arcuate nucleus and NPY neurons by fasting, diet-induced obesity, and 17beta-estradiol.* Mol Cell Endocrinol, 2016. **422**: p. 42-56.
68. Verhulst, P.-J. and I. Depoortere, *Ghrelin's second life: from appetite stimulator to glucose regulator.* World journal of gastroenterology, 2012. **18**(25): p. 3183-3195.
69. Park, S., et al., *Modification of ghrelin receptor signaling by somatostatin receptor-5 regulates insulin release.* Proceedings of the National Academy of Sciences of the United States of America, 2012. **109**(46): p. 19003-19008.
70. Licholai, J.A., et al., *Why Do Mice Overeat High-Fat Diets? How High-Fat Diet Alters the Regulation of Daily Caloric Intake in Mice.* Obesity (Silver Spring, Md.), 2018. **26**(6): p. 1026-1033.
71. Ibos, K.E., et al., *Kisspeptin-8 Induces Anxiety-Like Behavior and Hypolocomotion by Activating the HPA Axis and Increasing GABA Release in the Nucleus Accumbens in Rats.* Biomedicines, 2021. **9**(2).
75. Steyn, F.J., et al., *Development of a methodology for and assessment of pulsatile luteinizing hormone secretion in juvenile and adult male mice.* Endocrinology, 2013. **154**(12): p. 4939-45.
76. Schmittgen, T.D. and K.J. Livak, *Analyzing real-time PCR data by the comparative C(T) method.* Nat Protoc, 2008. **3**(6): p. 1101-8.
77. Yang, J.A., et al., *Stress rapidly suppresses in vivo LH pulses and increases activation of RFRP-3 neurons in male mice.* J Endocrinol, 2018. **239**(3): p. 339-350.
78. Negrón, A.L. and S. Radovick, *High-Fat Diet Alters LH Secretion and Pulse Frequency in Female Mice in an Estrous Cycle-Dependent Manner.* Endocrinology, 2020. **161**(10).
79. Qiu, J., et al., *Estradiol Protects Neuropeptide Y/Agouti-Related Peptide Neurons against Insulin Resistance in Females.* Neuroendocrinology, 2020. **110**(1-2): p. 105-118.
80. Roepke, T.A.; Zhang, C.; Rønnekleiv, O.K.; Kelly, M.J. *Gonadotropin-releasing hormone (GnRH) activates the m-current in GnRH neurons: An autoregulatory negative feedback mechanism?* Endocrinology **2008**, 149, 2459–2466.
81. Livak, K.J. and T.D. Schmittgen, *Analysis of relative gene expression data using real-time quantitative PCR and the 2(-Delta Delta C(T)) Method.* Methods, 2001. **25**(4): p. 402-8.
